# Genomic blueprints of sponge-prokaryote symbiosis are shared by low abundant and cultivatable *Alphaproteobacteria*

**DOI:** 10.1038/s41598-019-38737-x

**Published:** 2019-02-13

**Authors:** Elham Karimi, Tina Keller-Costa, Beate M. Slaby, Cymon J. Cox, Ulisses N. da Rocha, Ute Hentschel, Rodrigo Costa

**Affiliations:** 1 Faculty of Science and Technology, Algarve University, Gambelas, 8005-139 Faro Portugal; 2 Centre of Marine Sciences, Algarve University, Gambelas, 8005-139 Faro Portugal; 3 Institute for Bioengineering and Biosciences (iBB), Instituto Superior Técnico (IST), University of Lisbon, 1049-001 Lisbon, Portugal; 4 RD3 Marine Microbiology, GEOMAR Helmholtz Centre for Ocean Research Kiel, 24105 Kiel, Germany; 5 Department of Environmental Microbiology, Helmholtz Centre for Environmental Research - UFZ, 04318 Leipzig, Germany; 6 Christian-Albrechts-Universität zu Kiel, 24118 Kiel, Germany

## Abstract

Marine sponges are early-branching, filter-feeding metazoans that usually host complex microbiomes comprised of several, currently uncultivatable symbiotic lineages. Here, we use a low-carbon based strategy to cultivate low-abundance bacteria from *Spongia officinalis*. This approach favoured the growth of *Alphaproteobacteria* strains in the genera *Anderseniella*, *Erythrobacter*, *Labrenzia*, *Loktanella*, *Ruegeria*, *Sphingorhabdus*, *Tateyamaria* and *Pseudovibrio*, besides two likely new genera in the *Rhodobacteraceae* family. Mapping of complete genomes against the metagenomes of *S*. *officinalis*, seawater, and sediments confirmed the rare status of all the above-mentioned lineages in the marine realm. Remarkably, this community of low-abundance *Alphaproteobacteria* possesses several genomic attributes common to dominant, presently uncultivatable sponge symbionts, potentially contributing to host fitness through detoxification mechanisms (e.g. heavy metal and metabolic waste removal, degradation of aromatic compounds), provision of essential vitamins (e.g. B6 and B12 biosynthesis), nutritional exchange (especially regarding the processing of organic sulphur and nitrogen) and chemical defence (e.g. polyketide and terpenoid biosynthesis). None of the studied taxa displayed signs of genome reduction, indicative of obligate mutualism. Instead, versatile nutrient metabolisms along with motility, chemotaxis, and tight-adherence capacities - also known to confer environmental hardiness – were inferred, underlying dual host-associated and free-living life strategies adopted by these diverse sponge-associated *Alphaproteobacteria*.

## Introduction

Determining the ecological and evolutionary forces that shape the structure of marine sponge microbiomes is fundamental to current marine microbiology research due to the relevance of these symbiotic communities to ecosystem functioning^1–4^ and biotechnology^5–8^. Fifty-two bacterial phyla have been reported to inhabit sponges^3^, with *Proteobacteria* (mostly *Alpha-* and *Gammaproteobacteria*) being by far the most abundant, followed by *Acidobacteria*, *Actinobacteria*, *Chloroflexi*, *Nitrospirae*, *Cyanobacteria* and the candidate phylum *Poribacteria*^3,9^. Sponge-associated bacteria engage in nutritional exchange with their hosts and as such are considered to play an important role in benthic biogeochemical cycling^4,10,11^. Moreover, they are believed to produce most of the secondary metabolite repertoire of sponges^6,12–17^, and thus hold potential value for applications in medicine and pharmacy^7,16^.

*Alphaproteobacteria* display great versatility in their association with multicellular organisms, with interactions ranging from mutualistic over commensal to parasitic and pathogenic^18^. Microbial diversity surveys performed on different sponge species from various geographic locations have noted *Alphaproteobacteria* as regular sponge associates^9,19–21^. Particularly, a variety of currently uncultivatable lineages in the families *Rhodobacteraceae* and *Rhodospirillaceae* have been found as dominant members of the marine sponge microbiome^22–24^. Recent metagenomic “binning” studies, that sort metagenomic sequences into genomes that are assumed to constitute separate taxa, uncovered versatile metabolisms among diverse uncultivated *Alphaproteobacteria* symbionts of sponges, in which import and utilization of organic nitrogen and sulphur emerged as conspicuous features^2,25,26^. Among cultivated sponge-associated *Alphaproteobacteria*, the genera *Pseudovibrio* and *Ruegeria* likely rank as the best-described groups. They have been consistently isolated from various host species across the globe^19,20,27–29^, and based on recent genomic surveys, are considered to be well equipped for a symbiotic life-style^30–33^. In contrast, our understanding of the potential contribution of most cultivatable sponge-associated bacteria to holobiont functioning remains hindered by scarce knowledge of their genome content and architecture.

The known taxonomic diversity of sponge-derived culture collections is still limited, with 1% to 14% of the total sponge bacterial community estimated to be cultivatable using different methods^19,27,34,35^. Indeed, the most abundant bacterial symbionts of sponges, in particular, remain uncultivated^36,37^. Complicating factors for the cultivability of these bacteria are the initial sample processing method, the nature of the growth medium, and the incubation conditions^38^. The *in-situ* implantation of nutrient medium-containing diffusion growth chambers (DGCs)^39^ into sponge specimens and their subsequent incubation in the field^40^, or the concomitant use of several solid or liquid media (with and without antibiotics)^27,41^, for example, have been attempted to enlarge the phylogenetic breadth of marine sponge symbionts captured in the laboratory, and have shown promising results. However, continuous effort to cultivate hitherto “uncultivatable” symbionts or novel representative lineages within taxa less prone to cultivation is needed if we are to harness the metabolism of the marine sponge microbiome in a comprehensive fashion.

In this study, to attempt the isolation of “difficult-to-culture” bacterial symbionts of sponges - defined here as any organism detected in association with the sponge host regardless of whether the interaction is beneficial or obligatory^36^ - we used simple modifications to growth medium preparation and incubation conditions. First, we replaced the solidifying agent agar, which may inhibit the growth of certain bacterial taxa^42,43^, with the nontoxic agent gellan gum. In addition, to favour the cultivation of putatively slow-growing bacteria^44^, we prepared a low-carbon culture medium and utilized a lower incubation temperature (19 °C) with a prolonged incubation period (8 weeks). Our conditions favoured the cultivation of taxonomically diverse *Alphaproteobacteria* strains, especially of the genus *Ruegeria*, prompting us to (1) investigate the functional features of ten distinct genera spanning three *Alphaproteobacteria* orders (*Rhodobacterales*, *Sphingomonadales* and *Rhizobiales*) in detail, and (2) define the core functional attributes of *Alphaproteobacteria* species cultivated from the model sponge host *Spongia officinalis*. Cultivation-independent methods were employed to infer the relative abundance of the studied lineages in the *S*. *officinalis* microbiome, enabling us to critically contextualize the implications of genomic blueprints of symbiosis, identified across all these lineages, as possible factors enhancing host fitness.

## Material and Methods

### Sample collection, cultivation of bacteria, and phylogenetic analysis

Four *Spongia officinalis* specimens (Alg230-Alg233, for details see Karimi, *et al*.^23^) were collected in May 2014 by SCUBA diving at 20 m depth off the coast of Pedra da Greta (36° 58′ 47.2 N ;7° 59′ 20.8 W), southern Atlantic Ocean, Portugal, and transported to the laboratory within approximately 1 h in a cooling box. Specimens were processed immediately upon arrival: 2.5 g of the specimens’ inner body were cut and macerated with a sterile mortar and pestle in 22.5 mL of calcium and magnesium-free artificial seawater (CMFASW) (for details see Hardoim, *et al*.^45^; Esteves, *et al*.^29^). Suspensions were serially diluted in CMFASW after which 100 µL of 10^−3^ to 10^−8^ dilutions were spread on marine gellan gum medium (hereafter called ‘MG50’) plates in triplicates. ‘MG50’ was prepared by adding 0.802 g marine broth (MB; ROTH®) in 1 L artificial seawater (ASW) (MB 50 times diluted) and solidified with Phytagel™ (gellan gum; 5 gL^−1^). All plates were incubated for eight weeks at 19 °C. Bacterial growth was monitored weekly and colony forming units (CFUs) counted. Colonies were selected based on their variations in colour and shape with the aim of isolating as many different bacterial morphotypes as possible rather than randomly collecting a high number of strains. Nevertheless, highly abundant morphotypes were picked more often to enable the assessment of different bacterial lineages possibly sharing the same colony morphology (see Supplementary Table S1). Average CFU counts ranged from 3.0 × 10^6^ (specimen Alg232) to 8.1 × 10^6^ (specimen Alg230) CFUs g^−1^ sponge wet weight. Sponge specimen Alg231 had 6.9 × 10^6^ ± 0.00015 × 10^6^ CFUs g^−1^ (mean ± SE), and was chosen for colony isolation as it showed the greatest variety of morphologically distinct colonies. Here, we benefited from previous knowledge of the (equivalent) functional and taxonomic bacterial diversity present in each sponge specimen, acquired via shotgun metagenome sequencing^23^. This allowed us to calibrate our sampling effort to cover the total colony morphotype diversity within one specimen (higher morphotype sampling depth) rather than spreading the effort across several specimens, a strategy that would likely lead to the retrieval of the same and most abundant phylotypes from different specimens (lower morphotype sampling depth). In total, 48 colonies (many of which were morphologically unique) were picked and streaked to purity on MG50 plates. The purified isolates were then grown for 48 h in 1:2 diluted marine broth (MB2) and stocked in fresh MB2 supplemented with 20% glycerol at −80 °C. 16 S rRNA gene-based taxonomic affiliation of the isolates, from genomic DNA extraction to PCR amplification and classification using the RDP Classifier tool, were performed as established elsewhere^29,46^ and described in File S1 (Supplementary Information).

### Genome sequencing of sponge-associated *Alphaproteobacteria*

Although not specifically designed for this purpose, most of the isolates retrieved in our cultivation attempt belonged to the class *Alphaproteobacteria* (see below), leading us to inspect their coding potential through genome sequencing. In-depth 16S rRNA gene phylogenetic inference of the *Alphaproteobacteria* isolates obtained in this study was performed as detailed in File S1 (Supplementary Information) to select representative strains for comparative genomics. Thereafter, genomic DNA samples of ten phylogenetically distinct *Alphaproteobacteria* strains (representing all obtained *Alphaproteobacteria* genera) were sent for genome sequencing on an Illumina MiSeq platform at Mr. DNA (Shallowater, TX, USA). Paired-end libraries (2 × 301 bp) were generated and the genomes were assembled *de novo* into contigs with the NGen DNA assembly software by DNAStar, Inc. as described previously^47^. All contigs of each genome were subjected to a BLAST (NCBI) search via the computational cluster facility of the Algarve Centre of Marine Sciences (CCMAR). The extracted BLAST files were then analysed in MEGAN5^48^ to confirm whether the taxonomic affiliation of each contig matched that of its respective source strain. Contigs found not to fall within the expected taxonomic affiliation of its respective strain, and/or less than 1,000 bp in length, were discarded prior to annotation and downstream comparative analyses. Estimates of completeness and “contamination” of all genomes – as determined by the proportion of core single copy genes in each genome and their extent of duplication, respectively - were obtained using the CheckM tool, with lineage-specific marker sets selected at class, order, or family ranks^49^.

### Annotation and comparative analysis of genomes

Open Reading Frame (ORF) prediction and annotation of the genome sequences were performed using the RAST (Rapid Annotation using Subsystem Technology) prokaryotic genome annotation server (version 2.0) with standard procedures^50^. In addition, all genomes were uploaded to the software platform EDGAR 2.0^51^ where core and pan-genomes were defined and the number of singleton genes per genome was determined based on the coding sequences (CDSs) predicted using RAST. EDGAR was further employed to generate a phylogenomic tree and estimate average amino-acid and nucleotide sequence identities (AAI and ANI, respectively) for the ten *Alphaproteobacteria* genomes, following the approach of Karimi, *et al*.^26^. CDSs were also subjected to annotation based on Clusters of Orthologous Groups of Proteins (COGs) using the on-line server WebMGA (e-value = 0.001)^52^. Unless otherwise stated, quantitative functional comparisons between the genomes were performed using COG annotations after Hellinger transformation of COG profiles (i.e. square root calculation of the relative abundance of each COG entry in a given genome). To address the functional relatedness between the ten alphaproteobacterial genomes and determine whether statistically sound functional groups exist among strains, redundancy analysis (RDA) was performed with the software package Canoco 4.5 (Microcomputer power, Ithaca, USA). The “species fit range” function was applied using high stringent settings ( > 99%) to identify COGs exclusive to different functional groups. Where applicable (that is, when sample sizes – numbers of genomes - were not prohibitive) White’s non-parametric t-test was conducted within STAMP v2.0.9^53^ to identify COG entries differently abundant (i.e. “enriched” or “depleted”) between groups of genomes.

To identify core alphaproteobacterial functions deemed ecologically and evolutionarily informative in the context of sponge-bacteria symbiotic relationships, the lists of CDSs and COGs common to all genomes were manually inspected. Particularly, we looked for the presence of genomic signatures found to be markedly enriched or depleted in the *Spongia officinalis* endosymbiotic consortium^23,26^ or in marine sponges in general^12,54^ in the core genome of the *Alphaproteobacteria* strains cultivated and fully sequenced in this study. To test whether the relative abundance of the examined signatures varied significantly among functional genome groups (as determined by RDA, see above), one-way ANOVA, followed by a Tukey post-hoc test if significant, was performed after verifying that all data passed equal variance tests.

To gain further insight into their secondary metabolite production capacities beyond COG-based annotations, all genomes were screened for the presence of secondary metabolite biosynthetic gene clusters (BGCs) using antiSMASH v.3^55^.

### Relative abundance of sponge-associated *Alphaproteobacteria* across marine biotopes

Variations in coverage of each *Alphaproteobacteria* genome were inspected by mapping the already available microbial metagenomes from *S*. *officinalis* (four specimens), surrounding seawater (three replicates) and sediments (three replicates)^23^ against the assembled genome of each bacterium. To this end, the sequencing reads from the replicate metagenome samples within each marine biotope mentioned above were pooled and thereafter aligned to each *Alphaproteobacteria* genome using bowtie2 v. 2.2.6 at default settings^56^. The alignment scores, displayed as proportions of reads in the metagenomes that could be aligned with each single genome, were used as comparative measures of relative abundance of the studied alphaproteobacterial strains across *S*. *officinalis*, sediments and seawater. Additionally, genus-level inference of relative abundance in *S*. *officinalis*, seawater and sediment metagenomes was performed by calculating the proportion of protein-encoding genes (CDSs) assigned to the *Alphaproteobacteria* genera targeted in this study in each replicate metagenome sample. Genus-level taxonomic assignment of CDSs and subsequent relative abundance inference was achieved, in this study, using MG-RAST (Meta-Genome Rapid Annotation using Subsystems Technology) v3.0^57^ annotations made available previously for *S*. *officinalis*, seawater and sediment metagenomes (Karimi *et al*.^23^, MG-RAST study ID: 021215RCmetagenomes).

### Ethics statement

This study relied on *in situ* sampling of microorganisms from marine invertebrates without a nervous system, and as such was exempt from ethical approval procedures according to the current Portuguese legislation (Decreto-Lei n° 113/2013). This study did not occur within privately owned or protected areas. This study did not involve endangered or protected species. The sampling methodology privileged minimally invasive handling procedures, following the guidelines of the European Directive 2010/63/EU.

## Results

### Isolation and identification of *S*. *officinalis*-associated bacteria

**Figure 1:**
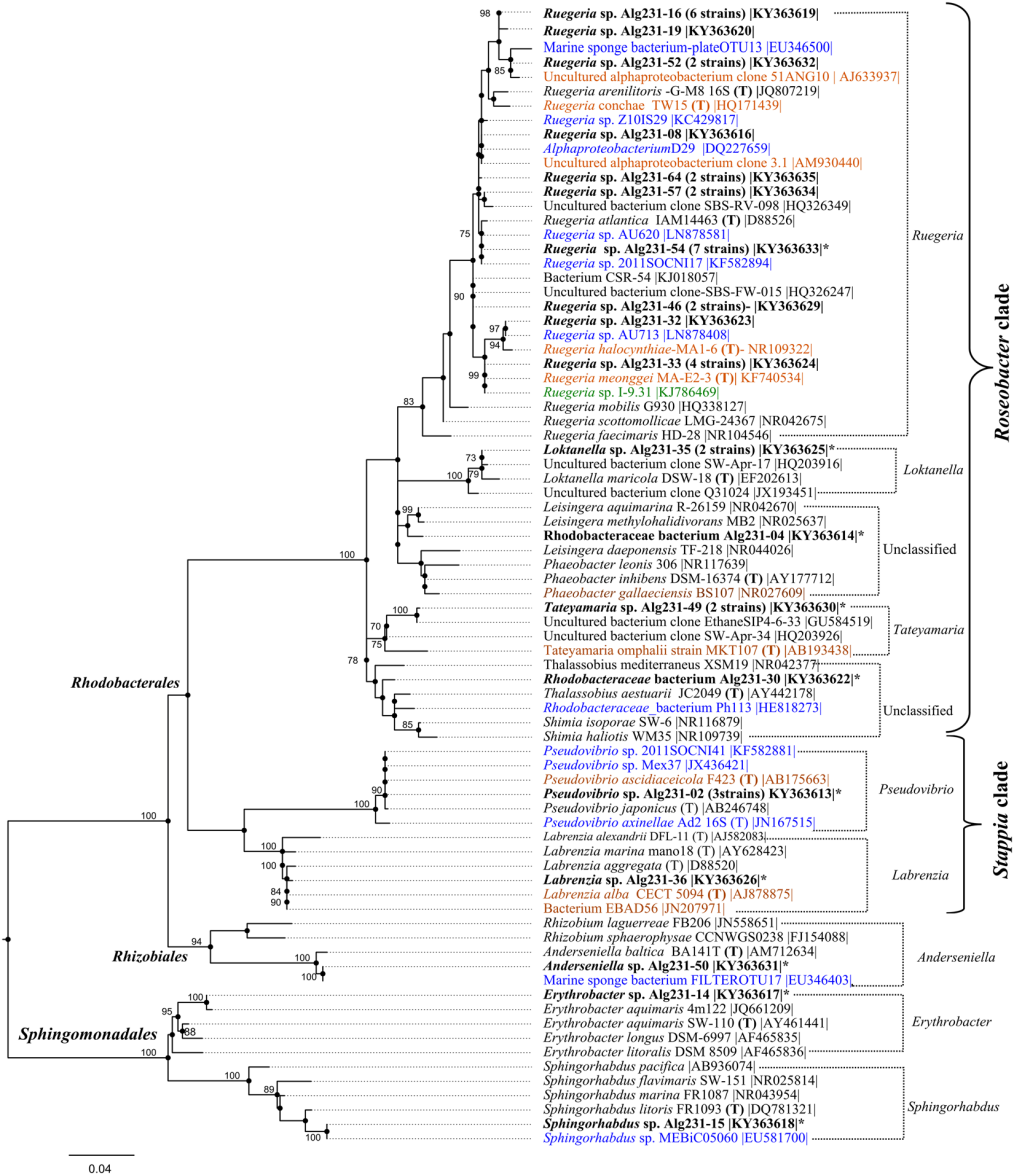
16S rRNA gene Maximum Likelihood tree of *Alphaproteobacteria* species. Kimura 2-parameter evolutionary distances between sequences were calculated using MEGA7^92^. *Alphaproteobacteria* strains isolated from *S*. *officinalis* are shown in bold, with each entry representing a unique OTU at 100% nucleotide homology cut-off. The number of isolates obtained from *S*. *officinalis* that belong to the same OTU are given in brackets. Closest NCBI BlastN hits and type strains (T) to each isolate are shown on the tree. Blue marks sponge-associated, orange marks invertebrate-associated and green marks marine algae-associated closest NCBI BlastN hits and type strains. Strains that had their genome sequenced are marked with an asterisk. Bootstrap values (500 repetitions) above 70% (0.7) are shown on the tree nodes. The tree contains 80 entries, and 682 nucleotide positions are included in the dataset.

Forty-eight aerobic, heterotrophic bacteria representing different colony morphologies were selected for further genotypic characterization (Supplementary Table S1), with 46 isolates belonging to the phylum *Proteobacteria* and only two isolates to the phylum *Actinobacteria* (Supplementary Table S2). Altogether, 12 formally recognized bacterial genera and two phylotypes unclassifiable at the genus level were identified, representing 24 unique 16S rRNA gene OTUs (Supplementary Table S2). Within the *Proteobacteria* isolates, the vast majority (41) affiliated with the *Alphaproteobacteria* class in the orders *Rhizobiales* (1 isolate), *Sphingomonadales* (2 isolates) and *Rhodobacterales* (38 isolates) (Fig. 1), whereas the remainder belonged to the *Gammaproteobacteria* class in the orders *Vibrionales* (4 isolates in the genus *Vibrio*) and *Alteromonadales* (1 isolate in the genus *Shewanella*) (Supplementary Tables S1 and S2). Among the *Alphaproteobacteria* isolates, two subgroups were represented within the *Rhodobacterales* order, namely the “*Roseobacter* clade”^58^ containing isolates classified as *Ruegeria*, *Loktanella*, *Tateyamaria* and two *Rhodobacteraceae* strains unclassifiable at the genus level (Alg231–04 and Alg231-30, see File S1 for details), and the “*Stappia* clade”^58^ containing isolates affiliated with the genera *Pseudovibrio* and *Labrenzia* (Fig. 1). Of the 19 unique OTUs assigned to the *Alphaproteobacteria*, ten belonged to the genus *Ruegeria* (see File S1 for details), while the remaining genera/phylotypes were represented by one single OTU each (Fig. 1). To determine the core genomic features of the diverse *Alphaproteobacteria* community cultivated from *S*. *officinalis*, we sequenced and compared the genomes of ten *Alphaproteobacteria* isolates representing eight formally accepted genera and two unclassifiable phylotypes (Alg231-04 and Alg231-30, Fig. 1, File S1) spanning the *Rhodobacteraceae*, *Rhodobiaceae*, *Sphingomonadaceae* and *Erythrobacteraceae* families (Table 1). The *Ruegeria* strain chosen for genome sequence represented the most abundant OTU within the genus (Fig. 1).

**Table 1:** Basic genome features of sponge-associated *Alphaproteobacteria* cultivated in this study.

Genomes	GC (%)	Size (Mbp)	Sequence depth (Gbp)	Genome coverage (x)	Contigs	Completeness (%)	Contamination (%)	Coding sequences (CDSs)	rRNAs	tRNAs	Accession numbers
*Anderseniella* sp. Alg231-50	57.9	4.61	0.65	143	8	100	0.22	4,635	3	42	LT703003-LT703010
*Erythrobacter* sp. Alg231-14	56.2	3.13	0.69	221	2	99.59	0.17	3,139	3	41	LT702999-LT703000
*Labrenzia* sp. Alg231-36	56.3	7.40	0.93	127	24	99.68	0.63	7,706	3	49	FREW01000001-FREW01000024
*Sphingorhabdus* sp. Alg231-15	52.8	3.62	0.47	132	2	99.14	0.79	3,702	3	42	LT703001-LT703002
*Pseudovibrio* sp. Alg231-02	51.3	5.96	0.73	124	26	99.94	0.05	5,674	12	65	FREX01000001-FREX01000026
*Rhodobacteraceae* bact. Alg231-30	55	4.54	0.76	169	10	99.25	0.32	4,604	6	43	FREU01000001-FREU01000010
*Rhodobacteraceae* bact. Alg231-04	59.6	4.81	0.95	198	29	99.20	0.32	4,784	11	50	FREY01000001-FREY01000029
*Ruegeria* sp. Alg231-54	56.5	4.92	0.76	155	35	99.25	0.11	5,120	6	44	FREZ01000001-FREZ01000035
*Loktanella* sp. Alg231-35	56.8	3.91	1.11	285	15	99.09	0.23	4,036	3	39	FREV01000001-FREV01000015
*Tateyamaria* sp. Alg231-49	57.4	4.51	0.77	173	39	99.68	0.80	4,793	3	38	FRFA01000001-FRFA01000039

### General features of the *S. officinalis*-associated *Alphaproteobacteria* genomes

The size of the assembled alphaproteobacterial genomes ranged between 3.13 Mb for *Erythrobacter* sp. Alg231-14 and 7.40 Mb for *Labrenzia* sp. Alg231-36. G + C contents varied from 51.3% in *Pseudovibrio* sp. Alg231-02 to 59.6% in the unclassified *Rhodobacteraceae* strain Alg231-04 (Table 1). The number of coding sequences ranged from 3,139 to 5,120 and the number of RNA genes from 41 to 77 including 3 to 12 ribosomal RNA (rRNA) genes (Table 1).

**Figure 2:**
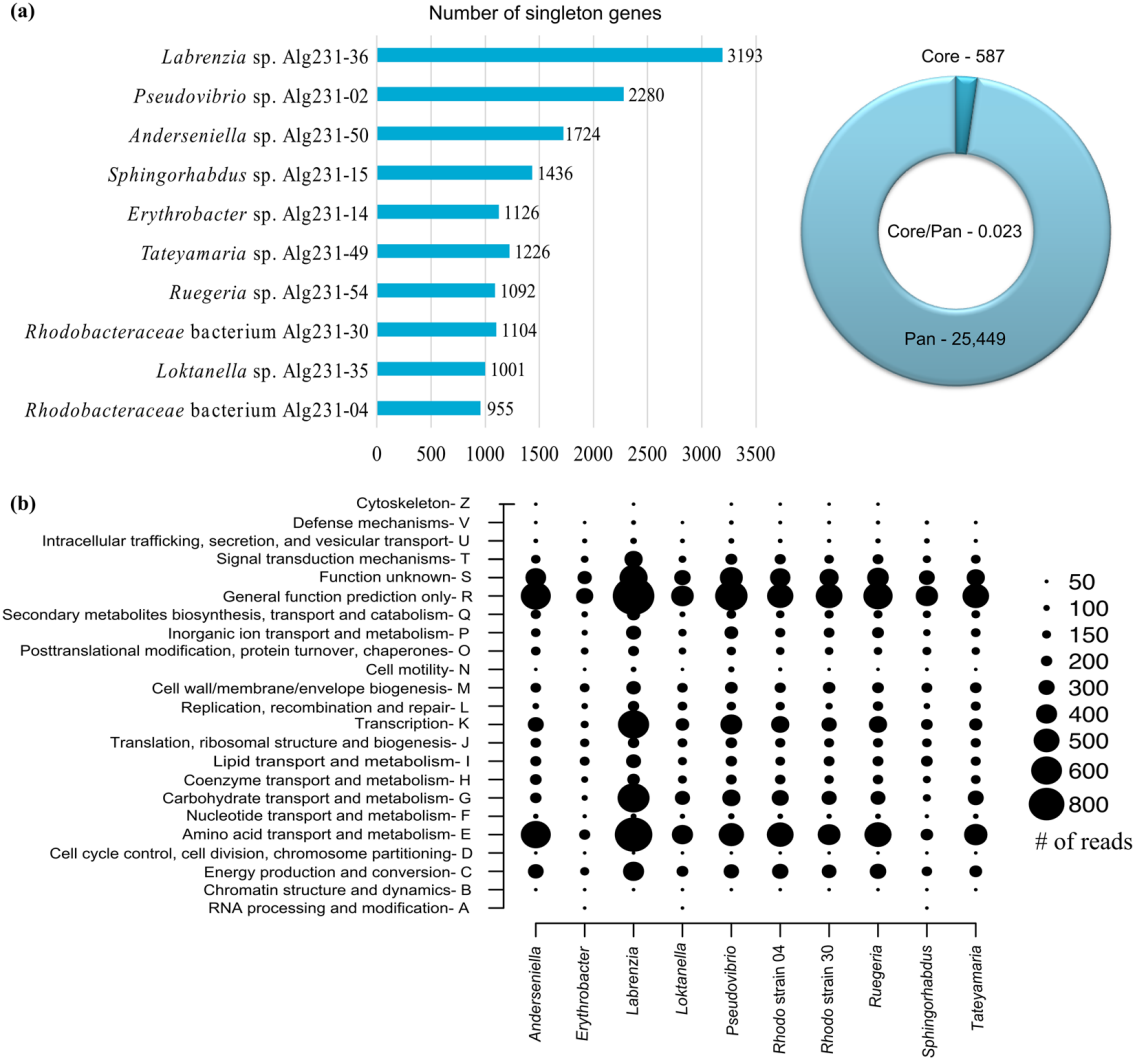
Singleton (i.e., strain-specific) genes, core and pan-genomes (**a**), and frequency plot of COG classes (**b**) across the *Alphaproteobacteria* genomes analysed in this study. The doughnut in (A) represents the core/pan-genome ratio retrieved from the dataset.

The core genome of the ten *S*. *officinalis* associated *Alphaproteobacteria* consisted of 587 genes (Supplementary Table S3, see below for details), while the pan-genome comprised 25,449 genes. The number of singleton genes unique to each genome ranged from 955 in *Rhodobacteraceae* bacterium Alg231-04 to 3,193 in *Labrenzia* sp. Alg231-36, and correlated to some extent with the phylogenetic position of the isolates: the five *Roseobacter* clade strains had the smallest numbers of singleton genes, followed by the *Sphingomonadales*, the *Rhizobiales* and then the two “*Stappia* clade” isolates *Pseudovibrio* Alg231-02 and *Labrenzia* Alg231-36, which as well possessed the largest genomes (Fig. 2a).

At a coarse level of functional resolution (i.e., COG classes), we found that the inspected strains possessed similar functional genome organization as COG classes ‘amino acid transport and metabolism’ (E), ‘transcription’ (K), ‘carbohydrate transport and metabolism’ (G), and ‘energy production and conversion’ (C), together with ‘general function prediction’ (R) and ‘function unknown’ (S), were the most dominant of the entire dataset (Fig. 2b). Although the rank distribution of COG classes differed somewhat between the individual genomes, the above-mentioned classes always prevailed compared to other classes, in each genome (Fig. 2b).

### Functional ordination of sponge-associated *Alphaproteobacteria* genomes

**Figure 3:**
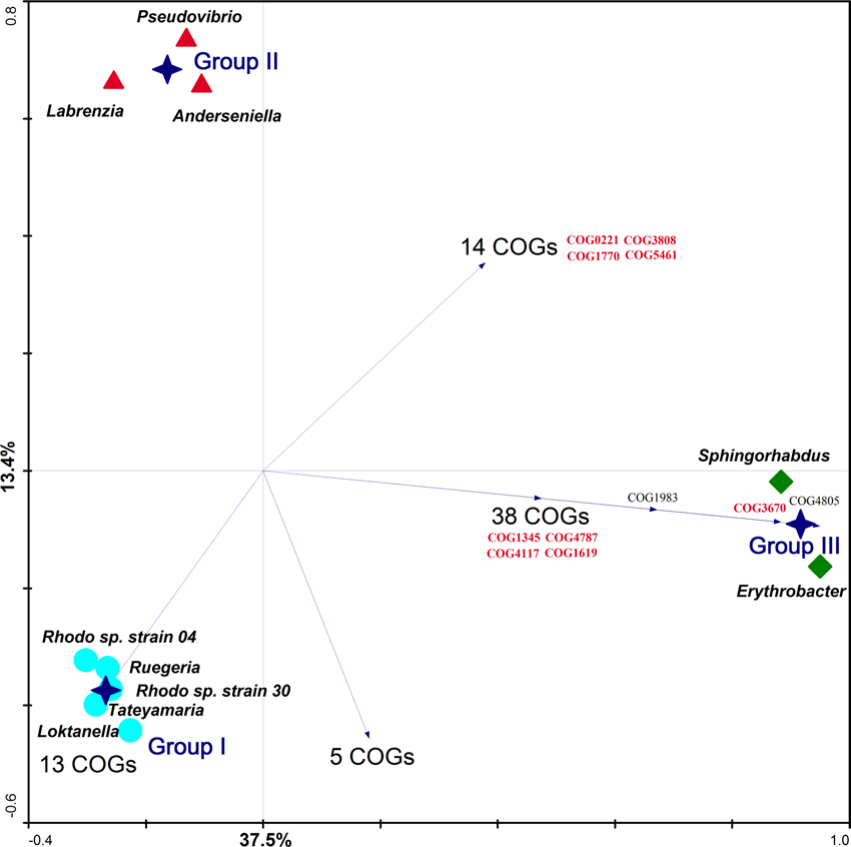
Functional ordination of *Alphaproteobacteria* genomes via Redundancy Analysis (RDA). Hellinger-transformed COG profiles were used as genome descriptors. Blue stars represent the centroid positions of functional genome Groups I, II and III, found to contribute significantly to variations in COG profiles as determined by Monte-Carlo permutation tests. Values displayed on the diagram axes refer to the percentage variation in the total dataset explained by the respective axis. Samples (i.e., genomes) are plotted in the ordination diagram in accordance with Euclidean distances calculated for each pair of genomes based on their COG relative abundance profiles. Arrows represent COGs displaying positive correlation fit > 99% with their corresponding genome group(s), all of which are listed in Supplementary Table S6. COGs highlighted in red have been approached more thoroughly in this study. Note the closer functional similarity between members of the “*Stappia* group” (*Pseudovibrio* and *Labrenzia*, formally belonging to the family *Rhodobacteraceae* in the order *Rhodobacterales*) to the genus *Anderseniella* (order *Rhizobiales*) (Group III) than to other genera of the *Rhodobacteraceae* family (Group I).

At the finest level of (COG-based) functional resolution, 2,804 individual COG entries were annotated in the ten genomes, with the number of COGs per genome ranging from 2,309 in *Erythrobacter* sp. Alg231-14 to 5,625 in *Labrenzia* sp. Alg231-36 (Supplementary Table S4) and 959 COG entries being shared by all genomes (Supplementary Table S5). Three functional groups were found to significantly contribute to variation in COG profiles after RDA: Group I, composed by the five *Roseobacter* clade genera/strains (*Rhodobacterales*); Group II, comprising *Anderseniella* sp. Alg231-50 (*Rhizobiales*) along with *Labrenzia* sp. Alg231-36 and *Pseudovibrio* sp. Alg231-02 (*Rhodobacterales*); and Group III, formed by *Erythrobacter* Alg231-14 and *Sphingorhabdus* Alg231-15 (*Sphingomonadales*) (Fig. 3). The clustering of Group II suggests that the genomes of *Labrenzia* and *Pseudovibrio* are functionally closer to that of *Anderseniella* than to other genera in the *Rhodobacterales* order*/Rhodobacteraceae* family (Group I) (Fig. 3), in line with genome-wide assessments of phylogeny^26,59^.

### Group-specific genome features

Figure 3 displays COG entries showing > 99% fit range with the above-mentioned functional genome groups, allowing us to quickly identify a suite of COGs (n = 73) (Supplementary Table S6), occurring in all genomes within one particular functional group and absent in the remainder. Within the 13 COGs found to be exclusive to Group I under this approach, seven could not be assigned a function and the remainders were involved with nutrient transport and metabolism, DNA replication and repair, and cell wall and ribosome biogenesis (Supplementary Table S6). In contrast, more ecologically informative COGs characterized functional groups II and III. Among these, we highlight COG1345 (Flagellar capping protein), COG1619 (Microcin C7 resistance protein MccF), COG3670 (lignostilbene dioxygenase), and COG4787 (Flagellar basal body rod protein), exclusive to Group III. Further, a protease II entry (COG1770, protein catabolism), two inorganic pyrophosphatases (COG0221; COG3808) important in lipid degradation and inorganic phosphate production, and a type IV pili component (COG5461) generally important for adherence, movement and host colonization - all traits usually regarded as host-associated adaptive features - were shared by Groups II and III while absent in Group I. To further determine characteristic genomic traits of the tightly clustering Group I (*Roseobacter* clade genomes), their COG profiles were, collectively, compared with those of the remaining five alphaproteobacterial genomes from Groups II and III using White’s non-parametric t-test (White *et al*., 2009). This quantitative comparison revealed 306 COGs differentially abundant between Group I and Groups II-III (Supplementary Table S6). COGs related to ABC transporters, sulphate/phosphate metabolism and secondary metabolite biosynthesis (COG class Q) were generally enriched in Group I, with N-acyl-L-homoserine lactone synthetases (COG3916) being typical of it. In contrast, predicted xylanase/chitin deacetylases (COG0726) and eukaryotic-like protein (ELP) COGs (COG0666, COG0790, and COG0457) were more abundant in functional genome Groups II-III (Supplementary Table S6, see also Table 2).

**Table 2:** COG entries involved in Restriction-Modification systems (V), Polyketide biosynthesis (Q), and Eukaryotic-like protein repeats (R) across the genomes of sponge-associated *Alphaproteobacteria* analyzed in this study.

COG	And.^1^	Ery.^2^	Lab.^3^	Lok.^4^	Pse.^5^	R.4^6^	R.30^7^	Rue.^8^	Sph.^9^	Tat.^10^	Class	Description
COG0286	0	0	0	0	1	1	1	0	0	2	V	Type I restriction-modification system methyltransferase subunit
COG0732	0	0	0	0	1	1	1	0	0	1	V	Restriction endonuclease S subunits
COG1002	0	0	0	0	0	1	0	0	0	0	V	Type II restriction enzyme, methylase subunits
COG1403	1	1	1	1	1	2	1	1	1	2	V	Restriction endonuclease
COG3440	0	0	0	0	1	2	0	0	0	1	V	Predicted restriction endonuclease
COG4096	0	0	0	0	0	0	1	0	0	0	V	Type I site-specific restriction-modification system, R (restriction) subunit and related helicases
COG3183	0	0	0	0	0	0	1	0	0	1	V	Predicted restriction endonuclease
COG3440	0	0	0	0	1	2	0	0	0	1	V	Predicted restriction endonuclease
COG3587	1	0	0	0	0	0	0	0	0	0	V	Restriction endonuclease
COG1002	0	0	0	0	0	1	0	0	0	0	V	Type II restriction enzyme, methylase subunits
COG1401	0	0	0	0	0	0	0	0	0	1	V	GTPase subunit of restriction endonuclease
COG3587	1	0	0	0	0	0	0	0	0	0	V	Restriction endonuclease
COG2761	1	1	2	2	1	1	2	2	1	3	Q	Predicted dithiol-disulfide isomerase involved in polyketide biosynthesis
COG3315	0	1	0	0	1	0	0	0	0	0	Q	O-Methyltransferase involved in polyketide biosynthesis
COG3319	0	0	1	1	2	0	2	1	0	1	Q	Thioesterase domains of type I polyketide synthases or non-ribosomal peptide synthetases
COG3321	0	0	3	1	2	0	1	0	0	0	Q	Polyketide synthase modules and related proteins
COG5285	5	1	3	3	4	4	3	4	3	5	Q	Protein involved in biosynthesis of mitomycin antibiotics/polyketide fumonisin
COG0666	3	1	1	1	2	0	0	1	1	0	R	Ankyrin repeats
COG0457	12	6	9	2	5	2	2	8	6	2	R	Tetratricopeptide repeats
COG2319	2	0	5	1	1	2	4	1	0	1	R	WD40 repeats
COG1520	0	2	1	1	1	1	1	1	1	1	R	WD40 repeats
COG4886	0	0	0	0	1	0	0	0	0	0	R	Leucine-rich repeat (LRR) protein
COG5424	1	0	0	0	0	0	0	0	0	0	R	pyrrolo-quinoline quinone repeat (PQQ)
COG0790	8	2	13	1	6	4	1	1	3	3	R	Tetratricopeptide repeats

COG Class V - “Defense mechanisms”; COG Class Q - “Secondary metabolites biosynthesis, transport and catabolism”; COG Class R - “General function prediction only” (**1**) *Anderseniella* sp. Alg231-50, (**2**) *Erythrobacter* sp. Alg231-14, (**3**) *Labrenzia* sp. Alg231-36, (**4**) *Loktanella* sp. Alg231-35, (**5**) *Pseudovibrio* sp. Alg231-02, (**6**) *Rhodobacteraceae* bacterium Alg231-04, (**7**) *Rhodobacteraceae* bacterium Alg231-30, (**8**) *Ruegeria* sp. Alg231-54, (**9**) *Sphingorhabdus* sp. Alg231-15, (**10**) *Tateyamaria* sp. Alg231-49.

### *Alphaproteobacteria* cultivated from *S*. *officinalis* share a versatile metabolism

#### Nutrient metabolism and cycling

All *Alphaproteobacteria* strains had several ABC-type transporter-encoding genes in common for the transport of sugars, dipeptides and branched-chain amino acids. Likewise, they possessed several copies of nitroreductase-encoding genes (COG0778) involved in the reduction of nitrogen-containing aromatic compounds. The nitrogen regulatory protein PII (COG0347), which mediates cell response to N availability, was also present with at least two gene copies in each genome, along with ammonia permease encoding genes (COG0004). Many important sulphur metabolic functions were common to the *Alphaproteobacteria* genomes. All contained several gene copies encoding for arylsulfatase A (COG3119), which breaks down sulphatides thus liberating sulphate, sulphate permeases (COG0659), taurine deoxygenase TauD (COG2175, taurine catabolism), sulphur transferases (COG2897), 3′-phosphoadenosine 5′-phosphosulfate (PAPS) 3′-phosphatase (COG1218; enzyme involved in sulphur assimilation and/or sulphate reduction) and sulphite reductases (COG0155; enzymes catalysing the reduction of sulphite (SO_3_^2−^) to hydrogen sulphide (H_2_S)). All strains also shared ABC-type components involved in phosphate transport (COG0226, COG1117, COG0573, COG0581), a phosphate uptake regulator (COG0704), and genes encoding for guanosine polyphosphate pyrophosphohydrolases/synthetases (COG0317).

We found that all genomes shared several genes encoding for proteins that contain or require B vitamins including thiamine (B1, COG0352), riboflavin (B2, COG0054; COG0307, COG1985), nicotinic acid (B3, COG1057), pyridoxamine phosphate oxidase (B6, COG0259), biotin (B7, COG0340), and cobalamin (B12, COG4547) (Supplementary Table S5). The presence of riboflavin synthase alpha and beta chains and of pyridoxal phosphate biosynthesis protein (PdxJ) encoding genes confirms the potential synthesis of vitamin B2 and B6 by all genomes.

#### *Defence*, *detoxification and antibiotic resistance*

All genomes possessed gene copies for cation and Na^+^ driven multidrug efflux pumps, ABC-type multidrug efflux systems and antimicrobial peptide transport systems (Supplementary Table S5). Hydrolases of the metallo-beta-lactamase superfamily and beta-lactamase class C were collective antibiotic resistance functions, whereby the *Sphingomonadales* strains *Sphingorhabdus* sp. Alg231-15 and *Erythrobacter* sp. Alg231-14 had the highest gene copy numbers with 16 and 11 genes, respectively. A gene encoding for an uncharacterized protein (COG1968) conveying resistance against the polypeptide antibiotic bacitracin was also detected (Supplementary Table S5). A shared catalase (peroxidase 1, COG0376) encoding gene could scavenge reactive oxygen species (ROS), while several glutathione S-transferase gene copies (COG0625) in each genome could aid in xenobiotic detoxification/oxidative stress. The genomes also harboured between one and three arsenate reductase-encoding genes for the reduction of arsenate to arsenite in arsenic detoxification processes. Moreover, all genomes were equipped with varied restriction-modification (R-M) systems (i.e. endonucleases) e.g. involved in anti-viral defence, but only one single R-M system (COG1403) was common to all of them (Table 2). Likewise, all genomes possessed genes involved in the biosynthesis of polyketides, but only one COG entry (COG5285 - mitomycin antibiotics/fumonisin) was shared by all genomes (Table 2).

#### Eukaryotic-like protein (ELP) encoding genes

Genes encoding for eukaryotic-like proteins (ELPs) usually thought to play a role in sponge-microbe interactions including ankyrin repeats (ANKs), tetratricopeptide repeats (TPRs), WD40 proteins, and pyrroloquinoline quinone (PQQ) were identified in all ten alphaproteobacterial genomes (Table 2). However, only *Anderseniella* sp. Alg231-50 possessed all the above-mentioned ELP types, and leucine-rich repeats (LRR) were detected only in *Pseudovibrio* sp. Alg231-02. Also, the *Anderseniella* strain, together with *Labrenzia* sp. Alg231-36, possessed the highest numbers of gene copies for the respective ELP motifs (Table 2).

#### Secondary metabolite gene clusters

**Figure 4:**
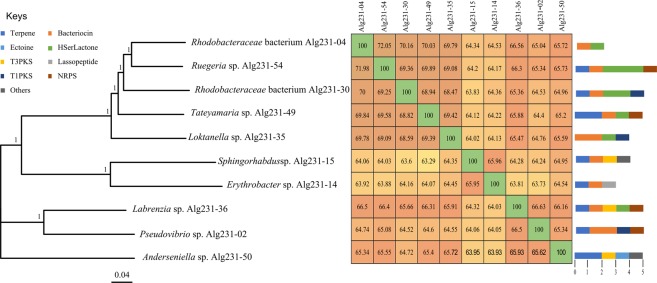
Phylogenomic tree and secondary metabolite biosynthesis potential of *Alphaproteobacteria* species cultivated from *Spongia officinalis*. The tree was generated using PHYLIP within the EDGAR environment. The neighbor joining method was applied on a matrix of Kimura distances between amino acid sequences predicted from all protein-encoding genes common to the ten genomes (core genes, n = 587). The scale bar represents the residue substitutions per site. Bootstrap values (300 repetitions) are shown on tree nodes. Sequence alignments were performed using MUSCLE. The heat-map shows the average nucleotide identity (ANI) calculated for each pair of genomes. Colored bars next to tree leaves represent gene clusters showing homology to known biosynthetic gene clusters (BGCs, see Supplementary Table S7 for details) after genome-wide screening with antiSMASH.

Using antiSMASH, all strains except *Rhodobacteraceae* bacterium Alg231-04 and *Erythrobacter* sp. Alg231-14 were found to harbour polyketide synthase (PKS)/non-ribosomal peptide (NRPS) gene clusters but, congruent with COG-based annotation (Table 2), diverged in terms of the diversity and types of compounds predicted to be produced (Fig. 4, Supplementary Table S7). Likewise, terpene synthase clusters were detected in eight of the ten strains but not in *Loktanella* sp. Alg231-35 and *Rhodobacteraceae* bacterium Alg231-04 (Fig. 4). Bacteriocin (peptidic toxins) gene clusters were detected for all cultivated strains except *Anderseniella* sp. Alg231-50. In contrast, only the *Anderseniella* strain harboured a gene cluster encoding for the osmolyte ectoine. Corroborating COG annotations, genes encoding for homoserine lactone signalling molecules were identified via antiSMASH in all *Roseobacter* clade genomes (Group I, Fig. 3) and, in addition, in *Labrenzia* sp. Alg231-36 (Fig. 4, Supplementary Table S7).

### Cultivatable *Alphaproteobacteria* share genomic blueprints of symbiosis

**Figure 5:**
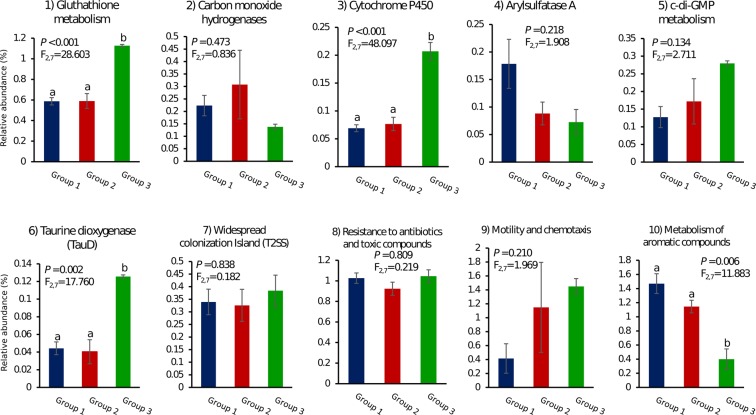
Relative abundance of ecologically informative genomic signatures across functional genome Groups I, II and III (Fig. 3). Columns represent average proportions (%) of genomic features in each functional group ± standard errors. The different letters above error bars indicate significant differences (P < 0.05) between groups. Respective F and P values are presented in the graphs. All data were of equal variance and all data except c-di-GMP-metabolism (5) and widespread colonization island (7) were normally distributed. COG annotations were used to infer the relative abundance of genomic features (1) to (7), determined by the ratio “total number of CDSs in the respective COG entry(ies)/total number of CDSs assigned to COGs” in each genome group. RAST annotations were used to infer the relative abundance of genomic features (8) to (10) (RAST subsystems), determined by the ratio “total number of CDSs in subsystem/total number of CDSs” in each functional group. Functions enriched (1–4, 6, 8, and 10) and depleted (5, 7 and 9) in the *S*. *officinalis* endosymbiotic consortium (Karimi *et al*., 2017), or in sponge-specific and uncultured *Alphaproteobacteria* lineages (Karimi *et al*., 2018), could be found across the genomes analysed in this study.

We investigated the relative abundance of all COG entries (COG-derived core genome, Supplementary Table S5) or CDSs (RAST-derived core genome, Supplementary Table S3) ascribed to functions present in the core genome that represent so-called “symbiosis factors” or “sponge-associated adaptive features” (e.g., Arylsulfatase A, Carbon monoxide hydrogenases – Cox, Cytochrome P450, resistance to heavy metals and xenobiotics) (Fig. 5). Some of the functions further explored (e.g. taurine metabolism, glutathione metabolism, resistance to antibiotics and metabolism of aromatic compounds) have been previously identified as enriched features of one hitherto uncultivatable, sponge-specific alphaproteobacterial lineage in the order *Rhodobacterales*^26^. Often no significant difference in COGs/CDSs relative abundance per functional genome group was found for the traits inspected, except for the quantitative enrichment of glutathione metabolism, taurine dioxygenase and cytochrome P450 encoding genes in Group III and the lower abundance of genes involved in the metabolism of aromatic compounds in the same group. Importantly, while displaying typical genomic features of several thus far uncultivatable sponge symbiotic bacteria, the here cultivated *Alphaproteobacteria* also possessed traits found to be “de-selected” in the *S*. *officinalis* endosymbiotic community in comparison with the surrounding environment^23,26^. These included, for instance, regulators of c-di-GMP metabolism along with Tad (Tight adherence) pilus- and motility and chemotaxis-encoding genes (Fig. 5).

### MG50 favours the cultivation of low-abundant sponge-associated *Alphaproteobacteria*

The available shotgun sequenced metagenomes of *S*. *officinalis*, seawater and sediment samples^23^ were mapped against the ten genomes sequenced in this study. The proportions of metagenome reads aligned to the target genomes were very low (Table 3). Under this strain-level approach, *Anderseniella* sp. Alg231-50 was the most dominant strain in the sponge metagenome (0.0082%) followed by *Labrenzia* sp. Alg231-36 (0.0068%) and *Ruegeria* sp. Alg231-54 (0.0067%), while *Rhodobacteraceae* bacterium Alg231-30 (0.0007%) clearly was the least abundant. All *Alphaproteobacteria* genome reads were somewhat more abundant in the seawater metagenome, followed by sediments and then *S*. *officinalis* (Table 3). As a frame of comparison with *Anderseniella* sp. Alg231-50, in this study 7x as many metagenomic reads from *S*. *officinalis* were found to align with the genome of the more dominant, uncultivated *Rhodospirillaceae* symbiont So9, reconstructed previously from the host’s microbial metagenome via genomic binning procedures^26^. Genus-level relative abundances calculated with the ratio “CDS reads assigned to taxon/total CDS reads in metagenome” returned higher percent values per taxon, but corroborated the trends of higher taxon representativeness in seawater, followed by sediments and then sponge metagenomes (File S1).

**Table 3:** Percent alignment of total metagenomic reads from *S*. *officinalis*, seawater and sediments with the genomes assembled in this study.

*Genome vs* metagenome	Aligned reads^a^	Percent (%)^b^	*Genome vs* metagenome	Aligned reads^a^	Percent (%)^b^
***Anderseniella*** **sp. Alg231-50**			***Ruegeria*** **sp. Alg231-54**		
*S*. *officinalis*	2610	0.00829	*S*. *officinalis*	2135	0.00678
Sediment	3264	0.01476	Sediment	6728	0.03043
Seawater	4846	0.0214	Seawater	7022	0.03101
***Erythrobacter*** **sp. Alg231-14**			***Sphingorhabdus*** **sp. Alg231-15**		
*S*. *officinalis*	1790	0.00568	*S*. *officinalis*	1708	0.00542
Sediment	2006	0.00907	Sediment	1794	0.00811
Seawater	4197	0.01854	Seawater	4050	0.01789
***Labrenzia*** **sp. Alg231-36**			***Tateyamaria*** **sp. Alg231-49**		
*S*. *officinalis*	2157	0.00685	*S*. *officinalis*	2008	0.00638
Sediment	2124	0.00961	Sediment	5485	0.02481
Seawater	4856	0.02145	Seawater	7380	0.03259
***Loktanella*** **sp. Alg231-35**			***Pseudovibrio*** **sp. Alg231-02**		
*S*. *officinalis*	1911	0.00607	*S*. *officinalis*	1776	0.00564
Sediment	2445	0.01106	Sediment	1709	0.00773
Seawater	7285	0.03217	Seawater	3913	0.01728
***Rhodobacteraceae*** **bact. Alg231-04**			***Rhodobacteraceae*** **bact. Alg231-30**		
*S*. *officinalis*	1987	0.00631	*S*. *officinalis*	236	0.00075
Sediment	3331	0.01507	Sediment	932	0.00422
Seawater	6326	0.02794	Seawater	2200	0.00972

The total number of paired-end sequence reads in the metagenome dataset (Karimi *et al*., 2017b) were as follows: *S*. *officinalis* −31,497,820; Sediment −22,107,730; Seawater −22,641,917.

^a^Aligned reads - the number of metagenomic sequence reads from a given environment that aligned with the genome sequence of the respective *Alphaproteobacterium* isolate.

^b^Percent (%) - the percentage of metagenomic sequence reads from a given environment that aligned with the genome sequence of the respective *Alphaproteobacterium* isolate.

## Discussion

To date most bacteria isolated from sponges have been affiliated with the phyla *Actinobacteria*, *Bacteroidetes*, *Firmicutes*, and *Proteobacteria*^29,35,60,61^. Our cultivation method (see File S1 for a detailed discussion) promoted the controlled growth of diverse *Alphaproteobacteria* species from *S*. *officinalis*, in line with the observations of Sipkema *et al*. (2011) who retrieved a majority of *Alphaproteobacteria* strains from *Haliclona* sp. with diverse oligotrophic media. Our bacterial isolation procedure placed sharp focus on distinct colony morphologies, enabling us to cultivate 14 bacterial genera within 48 cultures and to foster unprecedented, deep genome mining of putatively novel genera (Alg231-04 and Alg231-35, File S1), of the first-described genome sequence in the *Anderseniella* genus - found to possess many adaptive signatures for a symbiotic life-style (File S1), and of several other understudied lineages within the *Alphaproteobacteria* class. Indeed, only few genome assemblies are currently available on public databases for *Labrenzia* (22 assemblies), *Sphingorhabdus* (9), *Loktanella* (17), *Tateyamaria* (3), *Pseudovibrio* (24), *Ruegeria* (33), and *Erythrobacter* (89) species (our own assemblies included), in comparison with the number of genome assemblies available for intensively studied marine bacteria such as *Vibrio* spp. (2,798 genome assemblies). Moreover, although the abovementioned genera have already been cultivated before, most of the strains sequenced in this study (7 in 10) share less than 99 or 98% 16S rRNA gene similarity with the type strain of their closest, described species (Table S1). Finally, the genome-centred strategy employed in this study can be useful in solidifying the phylogeny of unresolved groups, which is likely the case of *Pseudovibrio* and *Labrenzia* strains and their placement within the *Alphaproteobacteria*^58,59^. It can also aid in the proposal of novel taxa (the case of strains Alg231-04 and Alg231-30) as supported, for instance, by genome-wide ANI/AAI estimates^62,63^.

It is possible that technical limitations such as insufficient metagenome sequencing depth and/or the usage of only short read lengths which may not align properly with reference genomes^64,65^ contribute to an underestimation of relative abundances calculated with the metagenome-genome mapping approach used in this study. Nevertheless, for all strains the percentages of aligned metagenome-genome reads were highest in seawater, followed by sediments and only then by sponge microbial metagenomes, a pattern corroborated by genus-level assessment of relative abundances using MG-RAST functional annotation (see File S1). Moreover, we verified that metagenome-genome mapping estimates delivered higher relative abundances for uncultivatable alphaproteobacteria representing dominant sponge symbionts. Altogether, these results indicate that (1) marine sponges are not the primary habitat of the here cultivated *Alphaproteobacteria* species, (2) bacterial culturing methods tend to sample rare members of the marine sponge microbiome (as suggested by Montalvo, *et al*.^66^ and extensively discussed by Hardoim, *et al*.^37^, (3) low-abundant sponge symbionts usually captured in culture evolve adaptive features that support a biphasic particle- (“free-living”)/host-associated life-style. Indeed, numerous “symbiosis factors” have been identified in the genomes of *Pseudovibrio* and *Ruegeria* spp., prompting extensive discussion on their potential roles in promoting host fitness^30,31,67^. The debate has, however, often disregarded the *in-situ* densities of the studied organisms, raising concerns about the net effect of the presumed adaptive features on holobiont functioning^68^. Evidence exists for the presence of these symbiosis factors in the genomes of both free-living and host-associated representatives of cultivatable sponge symbionts^68^, reinforcing the biphasic mode of living hypothesis, and such factors have been proposed to underlie the evolution of canonical commensal bacteria such as *Escherichia coli*^69^. Here, we delineate the core functional traits of sponge-associated alphaproteobacterial cultures and address their relevance as genomic hallmarks of symbiosis and bimodal life strategies.

In nutritional terms, the presence of arylsulfatase-encoding genes in all genomes, an attribute enriched in the marine sponge microbiome^23^ and revealed to be common among several uncultivated lineages of sponge symbionts^25,26^, underlies one possible role of this pool of *Alphaproteobacteria* species in consuming sulphated polysaccharides. The potential ability to break-down taurine, identified earlier as one adaptive feature of a currently uncultivatable and sponge-enriched *Rhodospirillales* clade (“SERC”^26^), was identified, in this study, among many low-abundance and cultivatable *Alphaproteobacteria* spp. In addition, several genes encoding for vitamin B biosynthesis were shared among our strains. Biotin (B7), thiamine (B1) and cobalamin (B12) biosynthesis capacities are common, for instance, among members of the *Roseobacter* clade^70^ and evidence exists for the participation of symbiotic *Alphaproteobacteria* in nourishing vitamins B1 and B12 required by a marine dinoflagellate (*Lingulodinium polyedrum*) for growth^71^. In line with this view, *Alpphaproteobacteria* spp. could likewise play an important role in providing essential nutrients for sponge growth and functioning.

Each of the studied isolates possessed hundreds of genes conferring resistance to antibiotics and toxic compounds. Particularly intriguing in this regard was the ubiquitous presence of genes encoding arsenate reductase that mediates the reduction of arsenate (As(V)) to arsenite (As(III)) in arsenic detoxification processes^72,73^. This feature has been recently assigned for the sponge symbiont *Entotheonella* sp. which mineralizes arsenic and barium in intracellular vesicles^74^. Furthermore, genes involved in ABC-type multidrug efflux systems, hydrolases of the metallo-beta-lactamase superfamily, and remediation of ROS stress (e.g. gluthathione metabolism genes) underline how versatile the mechanisms of cell detoxification employed by these organisms can be^75,76^. Such capabilities may substantially increase bacterial fitness within dense and chemically-rich microbial communities, and have been generally reported as distinguishing features of the marine sponge microbiome in cultivation-independent studies^12,23,26,54^.

All of the analysed alphaproteobacterial genomes have ELPs which are known sponge symbiosis factors because of the role they play in the modulation of cellular protein-protein interactions and in the prevention of symbiont phagocytosis by host cells^77,78^. Functional genome Groups II and III had altogether higher proportions of ELPs than Group I, suggesting higher affinity of members of the former groups in establishing favourable or more stable interactions with marine sponges. This seems to be particularly true for the *Anderseniella* and *Labrenzia* strains, which possessed the higher ELP counts among the surveyed genomes and showed the highest relative abundance values, respectively, in the *S*. *officinalis* microbiome. It remains to be determined whether ELPs could likewise be involved in bacterial adaptation to other marine hosts, supporting an emerging, generalist pattern of occurrence of cultivatable *Alphaproteobacteria* across multiple sessile invertebrates such as ascidians^79^, corals^80^, and bryozoans^81^. Particularly intriguing was also the presence of genes required for the tight-adherence (Tad) pilus secretion machinery in all strains. The Tad locus underlies the assembly of Flp (fimbrial low-molecular-weight protein) pili fundamental for cell aggregation, biofilm formation, surface attachment, host colonization and pathogenesis^82–84^. Along with protein domains known to mediate biofilm formation (e.g. EAL and CGDEF domains involved c-di-GMP metabolism) and a multitude of other cell motility and chemotaxis factors, the Tad locus equips their host cells not only with host-colonization aptitude but also environmental hardiness. All these genomic features were de-selected in the *S*. *officinalis* endosymbiotic consortium while being more pronounced, for instance, in sediment metagenomes^23^, suggesting that they might be more required for persistence in other microniches. We therefore posit that such traits have been subjected to purifying selection to favour the maintenance of a dual life-style among the studied organisms.

Using antiSMASH, we could detect several antibiotic biosynthetic gene clusters across the studied genomes, in line with accumulating *in vitro* evidence for mild to high antimicrobial activities by sponge-associated *Alphaproteobacteria* such as *Ruegeria*, *Pseudovibrio*, and *Labrenzia*^28,29,31,85–87^. Particularly, both terpene-synthase and polyketide-synthase (PKS) biosynthetic gene clusters were common among the studied strains, each being present in eight out of ten genomes, while COG annotations predicted PKS-encoding genes for all genomes. The roles and activities of polyketides from sponge symbiotic bacteria have been largely explored in the last fifteen years^6,13–15^, however much less is known about the potential contribution of bacterial symbionts as producers of terpenoids in marine sponges^23^. Intriguingly, terpenoid biosynthesis has been regularly documented in keratose marine sponges^61,88,89^, including *Spongia officinalis*^90^. Yet the origin of the biosynthesis (host or symbionts) has, to our knowledge, not been specifically addressed by regular chemical screening studies. Sponge-derived diterpenoids have shown antimicrobial activity against pathogenic bacteria such as *Pseudomonas aeruginosa*^88^. Dihydrogracilin A, a terpene extracted from *Dendrilla membranosa*, has been shown to possess immune modulatory and anti-inflammatory action^89^. In addition, except for *Anderseniella*, all other *Alphaproteobacteria* strains possessed the potential to produce bacteriocins commonly regarded to inhibit growth of closely related strains and, as such, considered to be major molecules shaping the structure of microbial communities *in situ*^91^. Our results reveal that polyketide, terpene and bacteriocin biosynthesis capacities, recently documented in several *Pseudovibrio* genomes^31,32^, are widespread across diverse sponge-associated *Alphaproteobacteria*, suggesting a pivotal contribution of this clade to the chemical complexity, natural product biosynthesis repertoire and taxonomic composition of the marine sponge microbiome.

In conclusion, the use of simple modifications to regular culture conditions coupled to dedicated genome-wide analysis of marine sponge symbionts enabled unprecedented access to highly versatile metabolisms across diverse understudied *Alphaproteobacteria*. To improve our capacity to domesticate the so-far uncultivatable portion of the marine sponge microbiome, the design of future culture media should consider our improved understanding of the nutritional requirements of these symbionts acquired via recent metagenomic binning studies^25,26^, which allow strain-level, deep insights into the physiology of uncultivated bacteria. Here, we disclose manifold genomic blueprints of the marine sponge microbiome^12,23,54^ across the genomes of several low-abundance, cultivatable symbionts of *Spongia officinalis*, providing support for the convergent evolution of symbiosis traits above the genus level within a class known for its widespread occurrence in association with sponge hosts, encompassing hundreds of cultivatable and so far uncultivable sponge-associated lineages^9,22,26^. Certainly, the genomic attributes revealed here are to be found among closely-related, cultivatable *Alphaproteobacteria* - as emphasized above for *Pseudovibrio* and *Ruegeria* strains - retrieved not only from sponges but also from other particle- and host-associated microniches, suggesting that such traits are widespread across diverse lineages of generalist marine bacteria. Taken together, the outcomes compiled here contribute to novel insights into the potential roles of alphaproteobacterial communities in mediating molecular interactions and shaping the structure of the marine sponge microbiome. They further open new opportunities for study regarding the roles of low-abundace microorganisms as consistent reservoirs of functional redundancy within nature’s microbiomes, likely promoting the resilience of host-associated microbial assemblages in the marine realm.

## Supplementary information

File S1 revised clean

Table S1

Table S2

Table S3

Table S4

Table S5

Table S6

Table S7

## Data Availability

The 16S rRNA gene sequences of the bacterial isolates were deposited at NCBI (https://www.ncbi.nlm.nih.gov/) under the accession numbers KY363613-KY363636. Assembled genome sequences reported in this study were deposited at the European Nucleotide Archive - European Molecular Biology Laboratory (ENA-EMBL - https://www.ebi.ac.uk/ena) under the study identification number PRJEB18465 (ERP020395). Genome sequence accession numbers are shown in Table 1. Results of all data analyses performed in this study are included in this published article (and its Supplementary Information files).
