# Supplementary material for: Genomic blueprints of sponge-prokaryote symbiosis are shared by low abundant and cultivatable Alphaproteobacteria
Source: Sci Rep. 2019 Feb 13;9:1999. doi: 10.1038/s41598-019-38737-x (PMC6374434; doi:10.1038/s41598-019-38737-x)
Supplement: Supplementary file 1 — File S1 revised clean [file 41598_2019_38737_MOESM1_ESM.docx]

***File S1 - Extended methodology, results and discussion***

**Genomic blueprints of sponge-prokaryote symbiosis are shared by low abundant and cultivatable *Alphaproteobacteria***

Elham Karimi^1,2^; Tina Keller-Costa^3^; Beate M. Slaby^4^; Cymon J. Cox^2^, Ulisses N. da Rocha^5^; Ute Hentschel^4,6^, Rodrigo Costa^2,3^

1. Faculty of Science and Technology, Algarve University, Gambelas 8005-139 Faro, Portugal.
2. Centre of Marine Sciences, Algarve University, Gambelas 8005-139 Faro, Portugal.
3. Institute for Bioengineering and Biosciences (iBB), Instituto Superior Técnico (IST), Universidade de Lisboa, 1049-001 Lisbon, Portugal.
4. RD3 Marine Microbiology, GEOMAR Helmholtz Centre for Ocean Research Kiel, 24105 Kiel, Germany.
5. Department of Environmental Microbiology, Helmholtz Centre for Environmental Research - UFZ. Permoserstr. 15, 04318 Leipzig, Germany.
6. Christian-Albrechts-Universität zu Kiel, 24118 Kiel, Germany.

**For correspondence:** Rodrigo Costa (rodrigoscosta@tecnico.ulisboa.pt)

**Methods**

***DNA extraction, taxonomic classification and phylogenetic inference of bacterial isolates***

For genomic DNA extraction, 2 mL aliquots of the shaken MB2 cultures were centrifuged at 10,000 g for 30 min. Genomic DNA was extracted from the resulting cell pellets using the Wizard® Genomic DNA Purification Kit (Promega, Madison, USA) according to the manufacturer’s instructions. Genomic DNA samples of all isolates were then subjected to 16S rRNA gene amplification and Sanger sequencing for identification as previously described ^1^. Closest matches to all sequence queries were identified using the BLAST algorithm (December 2016) of the National Center for Biotechnology Information (NCBI) ^2^. Taxonomic assignment of bacterial isolates to the genus level was performed using the classifier tool of the Ribosomal Database Project (RDP, release 11, ^3^ as described earlier ^1,4^. Closest 16S rRNA gene sequences from type strains were determined using the RDP sequence match tool. Operational taxonomic units (OTUs) at 100% sequence similarity were assigned by aligning all sequences using the ClustalW algorithm and by calculating a pairwise distance matrix in MEGA7 ^5^. To construct a phylogenetic tree comprising all *Alphaproteobacteria* (most abundant class of the collection) isolates obtained in this study, and thus more precisely infer which isolates could represent novel bacterial taxa, the 16S rRNA gene sequences of closest matches observed in BLAST searches and the respective closest *Alphaproteobacteria* type strains found in RDP were included in the alignment procedure. An appropriate evolutionary model was then determined using the ‘find best DNA models’ function of MEGA7. This was the Kimura 2-parameter model with a discrete gamma-distribution and invariable sites (*K2+G+I*). A Maximum Likelihood tree was then determined with bootstrap support using 100 repetitions (Figure 1).

**Results and discussion**

***Isolation and identification of Spongia officinalis-associated bacteria***

In total, 48 aerobic, heterotrophic bacterial isolates representing manifold colony morphologies were selected in this study for further genotypic characterization (Table S1), with 46 isolates belonging to the phylum *Proteobacteria* and two isolates to the phylum *Actinobacteria* (Table S2). Within the *Proteobacteria*, the vast majority of the isolates (41) affiliated with *Alphaproteobacteria*, while the remainder (5 isolates) was classified as *Gammaproteobacteria*. Isolates in the *Alphaproteobacteria* class encompassed three orders: *Rhizobiales, Sphingomonadales* and *Rhodobacterales*, the latter comprising most isolates (38 strains) (Figure1, Tables S1 and S2). Altogether, twelve formally-recognized bacterial genera and two phylotypes non-classifiable at the genus level were identified. Twenty-Eight *Rhodobacterales* isolates affiliated with the genus *Ruegeria* which was the most abundant genus of the collection and displayed a high degree of intra-generic diversity. Indeed, *Ruegeria* strains grouped into ten distinct OTUs (100% cut-off) across five different *Ruegeria* species (*R. arenilitoris, R. atlantica, R. conchae, R. halocynthiae* and *R. meonggei*). (Figure1). Overall, 24 unique 16S rRNA gene OTUs (at 100% sequence similarity cut-off) were observed across the data (Table S2). Many of the closest NCBI BLASTn hits and/or type strains to these OTUs originated from various marine sponge species or other invertebrate hosts including corals, bivalves, ascidians, squid and sea urchins. 16S rRNA gene phylogeny revealed that most of the isolates reported in this study affiliated with two subgroups within the *Rhodobacterales* order, namely the “*Roseobacter* group” containing isolates classified as *Ruegeria* sp.*, Loktanella* sp.*, Tateyamaria* sp. and *Rhodobacteraceae* (two strains, see below), and the “*Stappia* group” containing isolates affiliated with the genera *Pseudovibrio* and *Labrenzia* (Figure 1).

***16S rRNA gene phylogenetic inference of* Rhodobacteraceae sp*. strains Alg231-04 and Alg231-30***

Two isolates (Alg231-04 and Alg231-30) of the *Rhodobacteraceae* family were not classifiable at genus level (Figure1, Table S2) and likely represent at least novel bacterial species. Closest type strains *Phaeobacter inhibens* T5 and *Thalassobius aestuarii* JC2049 shared 98.1% and 97.8% 16S rRNA gene sequence similarity with strains Alg231-04 and Alg231-30, respectively, but phylogenetic analysis showed that these *S. officinalis* isolates clustered separately from both their closest type strains and other *Phaeobacter* (Figure FS1.1) and *Thalassobius* (Figure FS1.2) representatives. In fact, strains Alg231-04 and Alg231-30 grouped with other unclassified *Rhodobacteraceae* and/or uncultivated strains, leaving their genus-level taxonomic affiliation unresolved.

Moreover, strains belonging to the genera *Erythrobacter*, *Sphigorhabdus*, *Loktanella*, *Tateyamaria* and *Ruegeria* chosen for genome sequencing in this study showed 16S rRNA gene homologies lower than 99.0% to the type strain of their closest, described species (Table S1) suggesting they could represent novel species within these genera. In contrast, the 16S rRNA gene sequence of *Pseudovibrio* sp. strain 231-02, representing a well studied sponge-associated and cultivatable bacterium, shared 100% sequence similarity to *Pseudovibrio ascidiaceicola* (T) strain F423. Interestingly, both *Ruegeria* sp. strain 231-54 and *Pseudovibrio* sp. strain 231-02 showed 100% 16S rRNA gene homology with their respective closest NCBI BLASTn hits (Figure 1), which were retrieved from *S. officinalis* specimens sampled in the Mediterranean Sea ^6^.

**(B)**

| **231_04**  **(A)** | **Closest type strain (RDP, ≥1200bp); accession number** | **Similarity %** |
| --- | --- | --- |
| **1** | *Phaeobacter inhibens* strain T5 (T); (NR_042761) | 98.06 |
| **2** | *Phaeobacter gallaeciensis* strain BS107 (T); (NR_027609) | 97.98 |
| **3** | *Leisingera methylohalidivorans* strain MB2 (T); (NR_025637) | 97.69 |
| **4** | *Leisingera aquimarina* strain R-26159 (T); (NR_042670) | 97.57 |
| **5** | *Ruegeria scottomollicae* LMG 24367 (T); (AM905330) | 97.28 |
| **6** | *Leisingera caerulea* LMG 24369 (T); (AM943630) | 97.25 |
|  |  |  |
|  | **Closest hit on NCBI BLASTN; accession number** |  |
| **1** | *Rhodobacteraceae* bacterium ACEMC 26-3; (FM163007) | 99.88 |
| **2** | Uncultured bacterium clone OS3BR21; JN233117 | 99.42 |
| **3** | *Phaeobacter sp.* P97; (KX163077) | 98.68 |
| **4** | *Phaeobacter inhibens* strain DSM17395; ([CP002976](https://www.ncbi.nlm.nih.gov/nucleotide/398655788?report=genbank&log$=nucltop&blast_rank=2&RID=4325G2VK01R)) | 98.63 |
| **5** | *Phaeobacter gallaeciensis* strain 2.10; ([CP002972)](https://www.ncbi.nlm.nih.gov/nucleotide/398652061?report=genbank&log$=nucltop&blast_rank=3&RID=4325G2VK01R) | 98.63 |
| **6** | *Phaeobacter sp.* P104; (KX163079) | 98.61 |
| **7** | *Leisingera methylohalidivorans* strain MB2; ([NR_121711)](https://www.ncbi.nlm.nih.gov/nucleotide/659364630?report=genbank&log$=nuclalign&blast_rank=5&RID=4325G2VK01R) | 98.02 |
| **8** | *Phaeobacter* sp. strain 8-1; (AJ536670) | 97.61 |
|  |  |  |


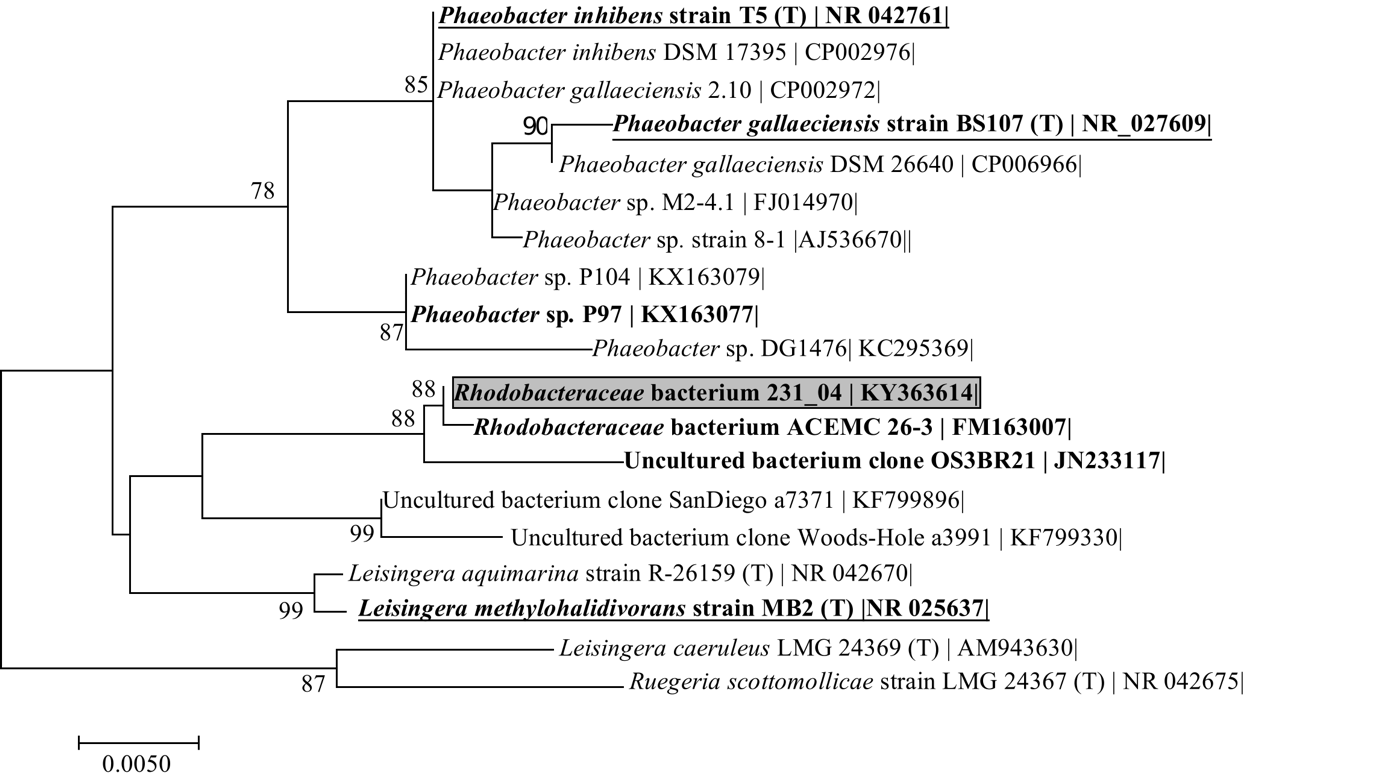


**Figure S1.1. (A)** The six closest type strains (T) (RDP Sequence match) and the eight closest NCBI BlastN hits to *Rhodobacteraceae* bacterium 231-04 are shown with their sequence similarity values. (**B)** 16S rRNA gene phylogeny of *Rhodobacteraceae* bacterium 231-04 (highlighted in grey) and close relatives based on the Maximum Likelihood method using the Kimura 2-parameter model. The top-three closest type strains (T) are highlighted in bold and underlined. The top-three closest NCBI BlastN hits are marked in bold. The tree with the highest log likelihood (-1688.3662) is shown. Five hundred replicates were run to bootstrap the tree. The percentage of trees in which the associated taxa clustered together is shown next to the branches (60% cut-off). A discrete Gamma distribution was used to model evolutionary rate differences among sites (5 categories (+*G*, parameter = 0.0558)). The rate variation model allowed for some sites to be evolutionarily invariable ([+*I*], 51.9478% sites). The tree is drawn to scale, with branch lengths measured in the number of substitutions per site. The analysis involved 19 nucleotide sequences. All positions containing gaps and missing data were eliminated. There were a total of 817 positions in the final dataset.

| **231_30**  **(A)** | **Closest type strain (RDP, ≥1200bp); accession number** | | **Similarity %** |  |
| --- | --- | --- | --- | --- |
| **1** | *Thalassobius aestuarii* JC2049 (T); (AY442178) | 97.8 | |  |
| **2** | *Shimia marina CL-TA03 (T);* (AY962292) | 97.69 | |  |
| **3** | *Thalassobius mediterraneus* CECT 5383 (T); (AJ878874) | 97.15 | |  |
| **4** | *Shimia haliotis* WM35 (T); (KC196071) | 97.14 | |  |
| **5** | *Leisingera aquimarina* LMG 24366T (T); (AM900415) | 96.49 | |  |
| **6** | *Leisingera methylohalidivorans* MB2 (T); (AY005463) | 96.06 | |  |
|  |  |  | |  |
|  | **Closest hit on NCBI BLASTN; accession number** |  | | |
| **1** | *Rhodobacteraceae* bacterium Ph113; ([HE818273](https://www.ncbi.nlm.nih.gov/nucleotide/391882036?report=genbank&log$=nucltop&blast_rank=1&RID=42XKAAYE015)) | 98.57 | |  |
| **2** | *Shimia sagamensis* strain JAMH 011; ([NR_137204](https://www.ncbi.nlm.nih.gov/nucleotide/1033657114?report=genbank&log$=nucltop&blast_rank=2&RID=42XKAAYE015)) | 98.35 | |  |
| **3** | *Rhodobacteraceae* bacterium 2tb2; ([FJ952817](https://www.ncbi.nlm.nih.gov/nucleotide/239740349?report=genbank&log$=nucltop&blast_rank=4&RID=42XKAAYE015)) | 98.13 | |  |
| **4** | Uncultured bacterium clone B12_10.3_2; ([FJ716880](https://www.ncbi.nlm.nih.gov/nucleotide/224714460?report=genbank&log$=nucltop&blast_rank=5&RID=42XKAAYE015)) | 98.13 | |  |
| **5** | Uncultured bacterium clone BF5_1108; (KC307193) | 98.02 | |  |
| **6** | *Alphaproteobacterium* C32; (AB302373) | 98.02 | |  |


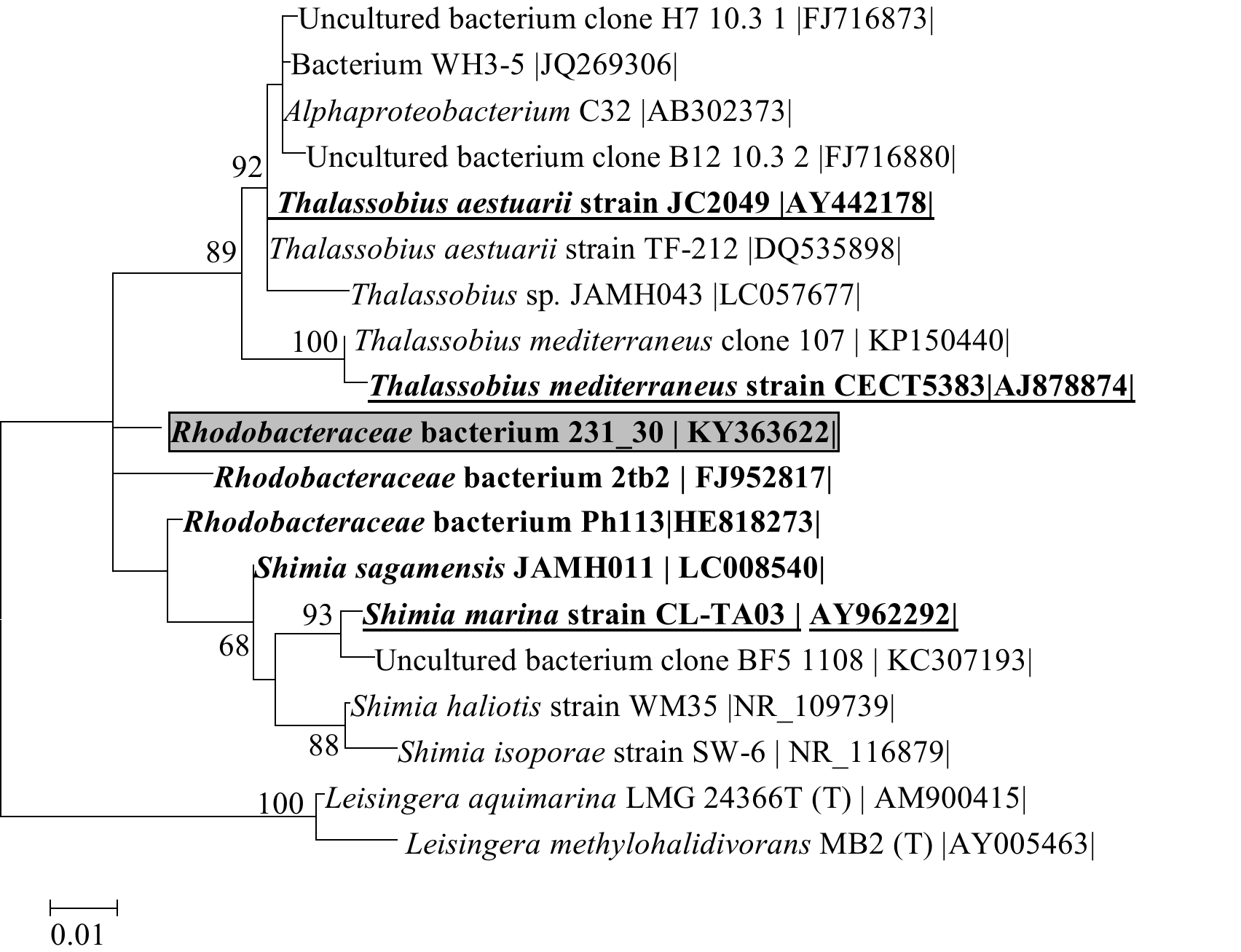


**(B)**

**Figure S1.2. (A)** The six closest type strains (T) (RDP Sequence match) and the six closest NCBI BlastN hits to *Rhodobacteracae* bacterium 231-30 are shown with their sequence similarity values. **(B)** 16S rRNA gene phylogeny of *Rhodobacteraceae* bacterium 231-30 (highlighted in grey) and close relatives based on the Maximum Likelihood method using the Tamura-Nei model. The top three closest type strains (T) are highlighted in bold and underlined. The top three closest NCBI BlastN hits are marked in bold. The tree with the highest log likelihood (-2050.0808) is shown. Five hundred replicates were run to bootstrap the tree. The percentage of trees in which the associated taxa clustered together is shown next to the branches (60% cut-off). A discrete Gamma distribution was used to model evolutionary rate differences among sites (5 categories (+*G*, parameter = 0.2182)). The rate variation model allowed for some sites to be evolutionarily invariable ([+*I*], 75.8138% sites). The tree is drawn to scale, with branch lengths measured in the number of substitutions per site. The analysis involved 19 nucleotide sequences. All positions containing gaps and missing data were eliminated. There were a total of 906 positions in the final dataset.

***Experimental design and cultivation strategy***

In this study, we used an oligotrophic medium along with low temperature and prolonged incubation time to attempt the cultivation of sponge–associated bacteria other than those usually retrieved with regular procedures (e.g. ^1,7-9^). We hypothesized that, on this basis, the emergence of diverse CFUs would be favored through the prevention of overgrowth by copiotrophic bacteria ^10^. Selecting isolates according to their distinctive morphological characters under the above-mentioned culturing conditions resulted in the retrieval of 14 bacterial genera within 48 cultures, enabling the cultivation of novel species and deep genome mining of several understudied bacterial lineages (e.g. *Anderseniella*, *Labrenzia*, *Sphingorhabdus*, *Loktanella*, *Tateyamaria*, besides unclassified lineages at the genus level). In comparison, for instance, Esteves and colleagues collected 279 strains with standard cultivation procedures (full strength marine agar medium and incubation at 25°C for three days) coupled to random, quantitative-based colony picking and purification, obtaining slightly more bacterial genera (17) despite the much larger cultivation effort ^1^. While the latter approach enables quantitative assessments of symbiont diversity and the analysis of genome diversification below species level, the methodology employed here led to the retrieval of a broad phylogenetic panel of isolates, enabling deep comparative genomic assessments among symbionts above the species level.

***Cultivatable sponge-associated* Alphaproteobacteria *display low abundances across marine biotopes***

While the metagenome-genome mapping procedure attempted in this study permits cultivation-independent inference of relative abundances at the strain level, the percent alignment values obtained were exceedingly low, and technical biases inherent to metagenome sequencing effort and read lengths preclude linear extrapolation of the data to infer actual abundances *in situ*. Although higher abundance rates were retrieved using MG-RAST taxonomic assignments based on the structure of protein-encoding genes across the metagenomes (Table FS1.1), this approach was otherwise limited to the genus level and is limited to taxonomic accuracy of the matching database for a sound diagnosis of relative abundances. For instance, this approach could not return any gene hit resembling the *Tateyamaria* and *Sphingorhabdus* genera, probably reflecting the low representativeness of these taxa in the database used or unsuitable alignment parameters in the search for somewhat divergent sequence reads. Therefore, it is felt that a deeper perspective of the true abundance of bacterial symbionts (both cultivated and uncultivated) in marine sponges is still required for a proper understanding of the relative forces exerted by several bacterial symbionts in holobiont functioning. This perspective cannot be achieved with the sole use of DNA sequencing technologies and, most likely, only a dedicated effort integrating taxon-oriented, high resolution imaging for cell visualization (enabled e.g. by fluorescent *in situ* hybridization coupled to confocal laser scanning microscopy, FISH-CLSM), symbiont cultivation, and deep microbiome sequencing may altogether enhance our understanding of symbiont abundance ranks in marine sponges, from the very dominant to the very rare bacterial associates. However, the methodologies employed here were congruent in establishing that (1) the alphaproteobacteria cultured in our study do not rank among the dominant symbionts of *S. officinalis* and (2) they are consistently more abundant in seawater and sediments than in *S. officinalis*. Clearly, the use of an oligotrophic medium based on synthetic seawater contributed decisively to this outcome, enabling us to delve deep into the genomics of low abundant and cultivatable marine bacteria.

**Table FS1.1** Relative abundances of alphaproteobacterial genera in *S. offciinalis* as inferred by the proportions of coding-sequence reads per metagenome assigned to each studied taxon.

| Genus | Sponges | Sediment | Seawater |
| --- | --- | --- | --- |
| *Anderseniella* | 1.9604E-05 ± 2.7122E-05 ^a^ | 0.00012482± 6.1781E-05^b^ | 1.8583E-05± 3.3804E-05^a^ |
| *Erythrobacter* | 0.01391589 ± 0.02704707^a^ | 0.20303477 ± 0.04607008^b^ | 0.08471498± 0.02181549^b^ |
| *Labrenzia* | 0.03913072 ± 0.06085259^a^ | 0.15229871± 0.00587699^b^ | 0.07239356 ± 0.00591571^b^ |
| *Pseudovibrio* | 0.01413875 ± 0.02621012^a^ | 0.04775088 ± 0.00568835^b^ | 0.01852317± 0.00041873^ab^ |
| Unclassified *Rhodobacteraceae* | 0.08684917 ± 0.04529446^a^ | 0.28537589 ± 0.09950285^b^ | 0.62217441± 0.04403614^b^ |
| *Ruegeria* | 0.33894996 ± 0.16368451^a^ | 1.04335701± 0.36604916^a^ | 1.85590135 ±0.09137426^ab^ |
| *Loktanella* | 0.02079868 ± 0.03852136^a^ | 0.069534 ± 0.0370023^ab^ | 0.36218096± 0.01101474^b^ |
| *Tateyamaria* | N.d | N.d | N.d |
| *Sphingorhabdus* | N.d | N.d | N.d |
| Shown are average percent values of CDS assigned to each genus in total metagenome CDS reads ± standard deviations, based on MG-RAST (M5rN) analysis. Values tagged with different letters are significantly different (p < 0.05) according to non-parametric ANOVA. N.d: not detected. | | | |

***Embedded description of the* Anderseniella *sp. Alg231-50 genome***

In this study, the genome of *Anderseniella* sp. Alg231-50 (Figure S1.3) was further explored for several reasons: first, of the here presented *Alphaproteobacteria* strains, Alg231-50 was the most dominant in the *S. officinalis* metagenome (Table 3). Second, many different types of eukaryotic like proteins (ELPs) were present in this genome, some of them even with high copy numbers, suggesting that Alg231-50 is well equipped for a symbiotic life-style. Third, to the best of our knowledge, there is no *Anderseniella* genome available on public databases yet (stand: 14^th^ of December 2018). Only eight genome sequences exist for the entire *Rhodobiaceae* family but none of them derived from a sponge host.

Of the 4,635 CDSs predicted in the *Anderseniella* genome using the RAST server, 1,494 were annotated as encoding for hypothetical proteins, but 4,109 CDSs could be assigned a COG function. Forty-five RNA genes were identified including 3 rRNAs and 42 tRNAs (Table 1). Quite remarkably, this strain shares several genome features in common with the sponge-specific, so-far unculturable *Rhodospirillaceae* symbiont So9 ^11^, including the potential to degrade aromatic hydrocarbons (e.g. toluene, biphenyl, benzoate, salicylate ester), tolerate heavy metals (copper, cobalt, mercury, chromium) and antibiotics (becta-lactams, fluoroquinolones, colicin E2), and utilize taurine and alkanesulfonates. Moreover, as observed for the *Rhodospirillaceae* symbiont *Spongia* So9 ^11^, RAST and COG annotations did not reveal any genes encoding for flagellar cell motility and chemotaxis in the *Anderseniella* genome (Table S4). Nevertheless, a type IV pili component (COG5461) and a protein required for attachment to host cells (COG5622) has been detected. The type IV pilus system (T4PS), is a multifunctional machine which, among other features, promotes adherence to eukaryotic cells ^12^. It has been shown that T4PS is important for pathogenic bacteria ^13^, for instance, to maximize biofilm formation upon host colonization ^14^. Overall, the presence of Tight Adherence (Tad) and Type IV adhesion proteins in the *Anderseniella* genome suggests eukaryotic host colonization aptitude consistent with other genomic traits identified in this organism to favor a symbiotic life-style. Furthermore, a highly versatile carbohydrate metabolism and the potential ability to synthesize polyphosphates were also inferred for strain Alg231-50. Nitrate (NO_3_^-^) transporter (COG0600, COG0715, COG1116, COG2223) and nitrate reductase (composing subunit alpha, beta, gamma and delta) (COG5013, COG1140, COG2181, COG2180 and COG3005) genes were as well observed (Table S4). These genes have been shown to modulate ammonia uptake and utilization via the general nitrogen regulatory system (Ntr) ^15^, suggesting a potential role of strain Alg231-50 in metabolic waste (that is, ammonia) removal thereby contributing to host fitness.

Additionally, the CRISPRfinder online tool was used with default settings to detect and identify CRISPR repeats and spacer sequences for the *Anderseniella* genome ^16^. Three possible CRISPR repeats with four spacers in total were predicted for this genome. CRISPRs are adaptive defense systems in bacteria which can memorize any attack from viruses and plasmids based on keeping conserved repeats and different spacer sequences ^17^. Metagenomic studies have shown that the marine sponge microbiome is enriched in CRISPR-Cas encoding genes ^18,19^ and, therefore, the presence of these elements in the *Andeseniella* genome is indicative of a close interaction between this symbiont and its sponge host.

The 16S rRNA gene of *Anderseniella* sp. strain Alg231-50 shares 100% similarity with that of “marine sponge bacterium strain FILTEROTU17”, isolated from *Haliclona* sp. ^20^. The closest described species to strain Alg231-50 is *Anderseniella baltica* (T) BA141, with 99.1% 16S rRNA gene identity (Table S1). Therefore, the genome sequence deposited in this study, besides representing the first entry for the genus *Anderseniella*, is most likely a representative of the species *Anderseniella baltica* based on 16S rRNA gene sequence homology. *Anderseniella* species have not been frequently reported from sponges, suggesting recalcitrance to common cultivation procedures. Finally, polyketide and terpene biosynthesis capacities (Table S7), traits found to be enriched in the microbial metagenome of *Spongia officinalis* ^19^ likely contribute to the secondary metabolite repertoire of the strain.

| 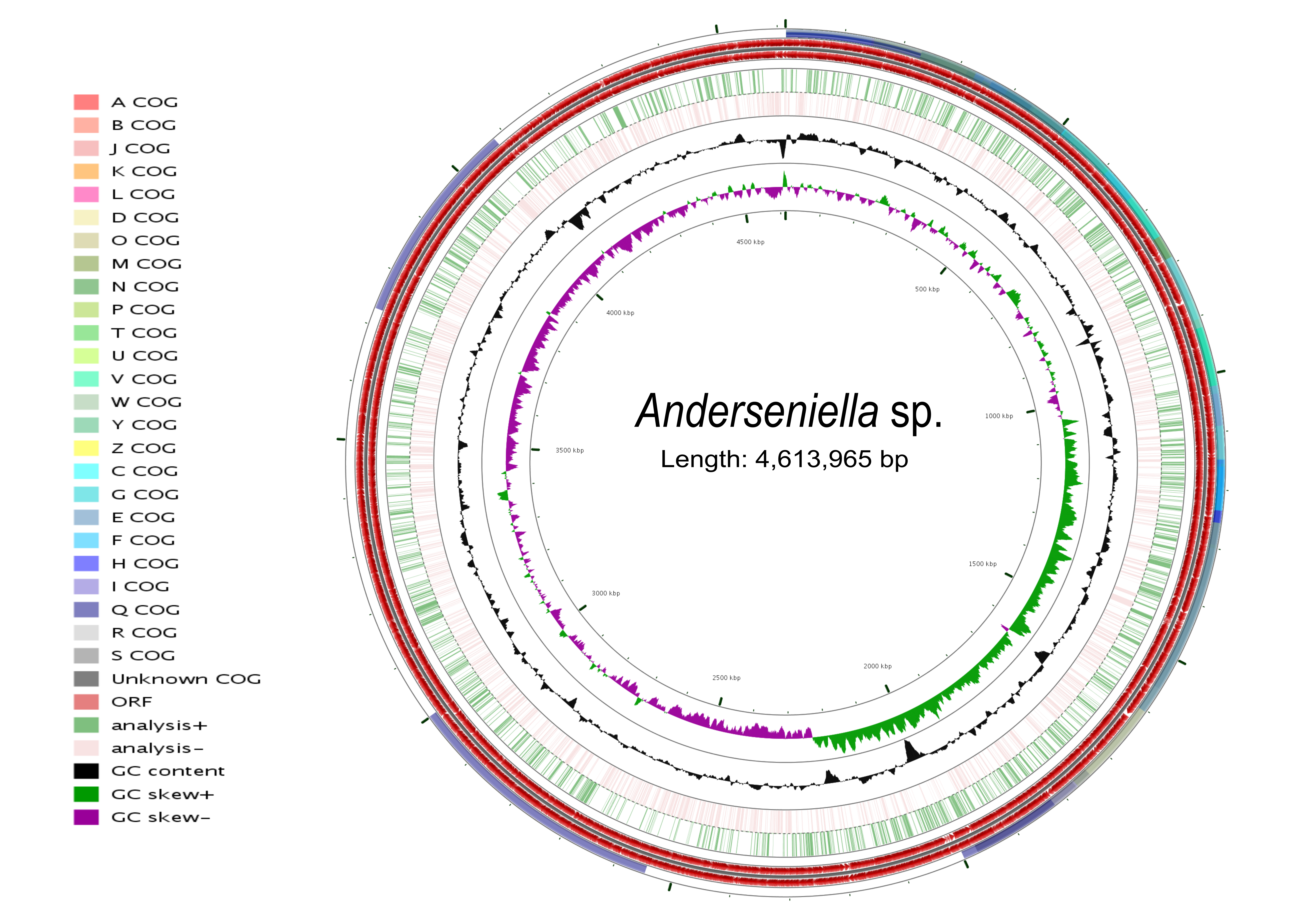 |
| --- |

**Figure FS1.3.** Genome map of *Anderseniella* sp. strain Alg231_50. The outer-most circle shows the distribution of COG classes across the genome, the second and third circles show the open reading frames (ORFs), the fourth and fifth circles display analysis scores for features (CDSs). The sixth circle shows the G+C% content plot (colored in black), and the inner-most circle the G+C skew (green and purple). The graphic was created on the CGView Server ^21^.

***COGs specific to and shared by functional genome clusters***

Functional genome clusters I, II and III, identified via ordination analysis based on COG profiles (Figure 3), shared 1,422 from the 2,804 COGs (50.7%) present in the whole dataset (Figure FS1.4). This is in line with the view of a considerable degree of functional conservation among the surveyed strains, which emerged from the more stringent analysis of 959 COGs (34.2%) common to all ten genomes. Clusters I and II further shared a much higher proportion of COGs than those exclusively shared by clusters I and III or II and III.


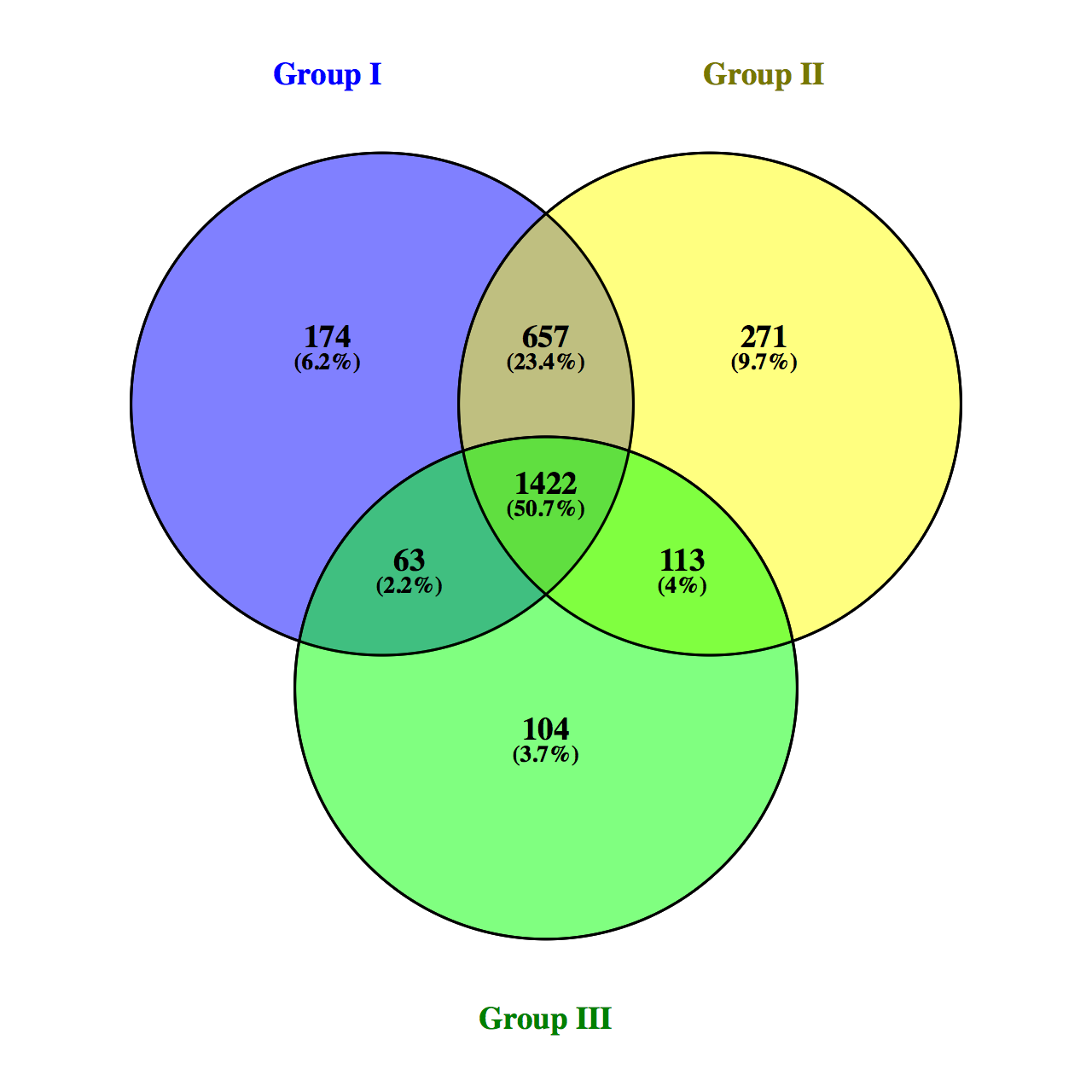


**Figure S1.4.** Venn diagram displaying COGs shared by and specific to functional genome clusters I, II and III. The diagram was created with Venny 2.1.0 online software ^22^.

**References**

1 Esteves, A. I. S., Hardoim, C. C. P., Xavier, J. R., Goncalves, J. M. & Costa, R. Molecular richness and biotechnological potential of bacteria cultured from Irciniidae sponges in the north-east Atlantic. *FEMS Microbiol Ecol* **85**, 519-536, doi:10.1111/1574-6941.12140 (2013).

2 Johnson, M. *et al.* NCBI BLAST: a better web interface. *Nucleic Acids Res.* **36**, W5-W9, doi:10.1093/nar/gkn201 (2008).

3 Cole, J. R. *et al.* The Ribosomal Database Project: improved alignments and new tools for rRNA analysis. *Nucleic Acids Res.* **37**, D141-D145, doi:0.1093/nar/gkn879 (2009).

4 Costa, R. *et al.* Evidence for selective bacterial community structuring in the freshwater sponge *Ephydatia fluviatilis*. *Microb Ecol* **65**, 232-244, doi:10.1007/s00248-012-0102-2 (2013).

5 Kumar, S., Stecher, G. & Tamura, K. MEGA7: Molecular Evolutionary Genetics Analysis version 7.0 for bigger datasets. *Mol Biol Evol*, msw054, doi:10.1093/molbev/msw054 (2016).

6 Bauvais, C. *et al.* Sponging up metals: bacteria associated with the marine sponge Spongia officinalis. *Mar Environ Res* **104**, 20-30, doi:10.1016/j.marenvres.2014.12.005 (2015).

7 Brinkmann, C., Kearns, P., Evans-Illidge, E. & Kurtbӧke, D. Diversity and Bioactivity of Marine Bacteria Associated with the Sponges Candidaspongia flabellata and Rhopaloeides odorabile from the Great Barrier Reef in Australia. *Diversity* **9**, 39, doi::10.3390/d9030039 (2017).

8 O’Halloran, J. *et al.* Diversity and antimicrobial activity of *Pseudovibrio* spp. from Irish marine sponges. *J Appl Microbiol* **110**, 1495-1508, doi:10.1111/j.1365-2672.2011.05008.x (2011).

9 Kennedy, J. *et al.* Isolation and Analysis of Bacteria with Antimicrobial Activities from the Marine Sponge Haliclona simulans Collected from Irish Waters. *Mar Biotechnol* **11**, 384-396, doi:10.1007/s10126-008-9154-1 (2009).

10 Thingstad, T. F. & Lignell, R. Theoretical models for the control of bacterial growth rate, abundance, diversity and carbon demand. *Aquat Microb Ecol* **13**, 19-27, doi:10.3354/ame013019 (1997).

11 Karimi, E. *et al.* Metagenomic binning reveals versatile nutrient cycling and distinct adaptive features in alphaproteobacterial symbionts of marine sponges. *FEMS Microbiol Ecol* **94**, fiy074-fiy074, doi:10.1093/femsec/fiy074 (2018).

12 Burrows, L. L. Prime time for minor subunits of the type II secretion and type IV pilus systems. *Mol Microbiol* **86**, 765-769, doi:10.1111/mmi.12034 (2012).

13 Melville, S. & Craig, L. Type IV Pili in Gram-Positive Bacteria. *Microbiology and Molecular Biology Reviews : MMBR* **77**, 323-341, doi:10.1128/MMBR.00063-12 (2013).

14 Varga, J. J., Therit, B. & Melville, S. B. Type IV Pili and the CcpA Protein Are Needed for Maximal Biofilm Formation by the Gram-Positive Anaerobic Pathogen Clostridium perfringens. *Infect. Immun.* **76**, 4944-4951, doi:10.1128/IAI.00692-08 (2008).

15 Moreno-Vivián, C., Cabello, P., Martínez-Luque, M., Blasco, R. & Castillo, F. Prokaryotic nitrate reduction: molecular properties and functional distinction among bacterial nitrate reductases. *J Bacteriol* **181**, 6573-6584 (1999).

16 Grissa, I., Vergnaud, G. & Pourcel, C. CRISPRFinder: a web tool to identify clustered regularly interspaced short palindromic repeats. *Nucleic Acids Res.* **35**, W52-W57, doi:10.1093/nar/gkm360 (2007).

17 Barrangou, R. & Marraffini, L. A. CRISPR-Cas systems: prokaryotes upgrade to adaptive immunity. *Mol Cell* **54**, 234-244, doi:10.1016/j.molcel.2014.03.011 (2014).

18 Horn, H. *et al.* An enrichment of CRISPR and other defense-related features in marine sponge-associated microbial metagenomes. *Front Microbiol* **7**, 1751, doi:10.3389/fmicb.2016.01751 (2016).

19 Karimi, E. *et al.* Comparative Metagenomics Reveals the Distinctive Adaptive Features of the *Spongia officinalis* Endosymbiotic Consortium. *Front Microbiol* **8**, doi:10.3389/fmicb.2017.02499 (2017).

20 Sipkema, D. *et al.* Multiple approaches to enhance the cultivability of bacteria associated with the marine sponge Haliclona (gellius) sp. *Appl Environ Microbiol* **77**, 2130-2140, doi:10.1128/AEM.01203-10 (2011).

21 Grant, J. R. & Stothard, P. The CGView Server: a comparative genomics tool for circular genomes. *Nucleic Acids Res.* **36**, W181-W184, doi:10.1093/nar/gkn179 (2008).

22 Oliveros, J. C. VENNY, An interactive tool for comparing lists with Venn diagrams. <http://bioinfogp.cnb.csic.es/tools/venny/index.html> (2007-2015).
